# Off-Clamp Versus On-Clamp Robot-Assisted Partial Nephrectomy: A Meta-Analysis of Clinical Trials and Matched Cohort Studies

**DOI:** 10.7759/cureus.97538

**Published:** 2025-11-23

**Authors:** Abdelrahman Abdelhameed, Ahmad Ali, Salma Ahmed, Nada Ramadan, Farzana Haque, Aranee Thirukketheesparan, Maria Khan, Moustafa Elattar, Shahida A Siddik, Ahmet Suleyman, Mohamed Hesham Gamal

**Affiliations:** 1 General Medicine, Warwick Hospital, South Warwickshire University NHS Foundation Trust, Warwick, GBR; 2 Orthopedic Surgery, Faculty of Medicine, Minia University, Minia, EGY; 3 General Practice, Faculty of Medicine, Mansoura University, Mansoura, EGY; 4 Biochemistry, Benha University, Benha, EGY; 5 Neurology, Barking, Havering and Redbridge University Hospitals NHS Trust, London, GBR; 6 Neurology, Queen's Hospital, Barking, Havering and Redbridge University Hospitals NHS Trust, London, GBR; 7 Acute Medicine, University Hospital of North Tees, Stockton-on-Tees, GBR; 8 General Practice, Faculty of Medicine, Benha University, Benha, EGY; 9 Acute Medicine, Whipps Cross University Hospital, London, GBR; 10 Internal Medicine, Heartlands Hospital, University Hospitals Birmingham NHS Foundation Trust, Birmingham, GBR; 11 Pharmacy, Benha University Hospital, Benha, EGY; 12 Pharmacology and Therapeutics, Faculty of Pharmacy, Tanta University, Tanta, EGY

**Keywords:** meta-analysis, minimally invasive surgery, off-clamp technique, on-clamp technique, renal cell carcinoma, robot-assisted partial nephrectomy

## Abstract

Kidney cancer incidence continues to rise globally, with partial nephrectomy established as the standard of care for localized renal masses. Robot-assisted partial nephrectomy has become the preferred approach, with debate continuing regarding the optimal management of renal blood flow during tumor excision. While on-clamp techniques involve temporary renal artery occlusion to minimize blood loss, off-clamp approaches avoid ischemic injury by maintaining continuous renal perfusion.

This meta-analysis aimed to compare perioperative, functional, and oncological outcomes between off-clamp and on-clamp robot-assisted partial nephrectomy. A systematic review was conducted following the Preferred Reporting Items for Systematic Reviews and Meta-Analyses (PRISMA) guidelines, searching PubMed, Web of Science, Scopus, and Cochrane databases. Twenty studies, comprising two randomized controlled trials and 18 observational studies, were included, totaling 4,961 patients after matching. Quality assessment utilized the Newcastle-Ottawa Scale for observational studies and the Cochrane Risk of Bias 2 tool for randomized trials. Meta-analysis employed risk ratios (RRs) for dichotomous outcomes and mean differences (MDs) for continuous variables, with random-effects models applied when heterogeneity exceeded 50%. Publication bias was assessed using funnel plots and Egger's regression test. Off-clamp robot-assisted partial nephrectomy demonstrated significantly lower major complication rates compared to on-clamp techniques (RR: 1.66, p = 0.018). Renal function preservation favored the off-clamp approach, with better postoperative glomerular filtration rate (MD: -3.45 mL/min/1.73 m^2^, p < 0.0001). The on-clamp technique showed less estimated blood loss (MD: -32.7 mL, p < 0.0001), though this did not translate to differences in transfusion requirements (RR: 0.80, p = 0.208). Operative time was longer for off-clamp procedures (MD: 21.55 minutes, p = 0.030). Hospital stay duration did not differ significantly between techniques (MD: 0.02 days, p = 0.946). Subgroup analysis by follow-up duration revealed that renal function benefits were most pronounced at intermediate (three to nine months) and long-term (≥12 months) follow-up.

Our study concluded that off-clamp robot-assisted partial nephrectomy offers superior safety, with significantly reduced major complications and better preservation of renal function, particularly at intermediate and long-term follow-up, despite requiring longer operative time. Both techniques demonstrate equivalent oncological efficacy with comparable positive surgical margins. The off-clamp approach should be preferred for patients at high risk of ischemic injury. At the same time, technique selection should be individualized based on patient renal reserve, tumor complexity, and surgical expertise.

## Introduction and background

Kidney cancer represents a significant public health concern, with the American Cancer Society projecting approximately 81,800 new cases and nearly 15,000 deaths in 2023 [1]. The incidence has been rising steadily over the past two decades at an annual rate of roughly 2% per year [2]. Kidney cancer incidence and mortality vary by region, age, and gender, with the highest rates currently observed in developed countries. However, recent trends indicate declining mortality in some Western European nations [3].

The majority of renal masses require surgical intervention as the definitive curative treatment; the optimal surgical approach remains a critical consideration. Partial nephrectomy (PN) has emerged as the standard of care for the management of localized renal masses. PN is increasingly favored over radical nephrectomy for the management of localized renal masses due to its nephron-sparing advantages. PN offers favorable oncological outcomes while preserving nephron mass and minimizing the risk of progression of chronic kidney disease (CKD) [4,5]. Preserving renal function is critical because postoperative kidney function decline increases the risk of cardiovascular disease, reduces quality of life, and may necessitate dialysis in severe cases.

With the advancement of minimally invasive surgical techniques, robot-assisted partial nephrectomy (RAPN) has become the preferred modality for the excision of small renal masses, demonstrating superior morbidity profiles compared to open or laparoscopic approaches [6]. The utilization of RAPN has increased substantially over the past decade, with studies reporting that robotic approaches now account for over 50% of all PNs performed in the United States, reflecting its growing acceptance in urological practice [7].

During RAPN, hilar clamping of the renal artery is a widely employed intraoperative technique to minimize blood loss during tumor resection and enhance visualization for precise dissection. This approach, known as the on-clamp technique, involves temporarily occluding renal blood flow during tumor excision [8]. However, warm ischemia time (WIT), which represents the period of renal artery occlusion, is a recognized independent predictor of postoperative renal function decline and unfavorable perioperative outcomes [9].

The primary mechanisms contributing to renal function impairment following PN include nephron loss from tissue resection and incomplete or failed recovery of nephrons due to ischemic injury from temporary blood flow interruption [10]. When blood flow is restored after clamping, ischemia-reperfusion injury occurs, characterized by oxidative stress, inflammation, and microvascular dysfunction, which can cause permanent nephron loss [10]. To mitigate the potential adverse effects of warm ischemia on renal function, off-clamp RAPN has been proposed as an alternative technique to the traditional on-clamp approach [11].

While previous systematic reviews and meta-analyses have mainly demonstrated comparable perioperative outcomes between the two techniques, there remains insufficient evidence to definitively establish the superiority of one approach over the other with respect to renal function preservation [12,13]. Specifically, insufficient evidence exists to definitively prove which approach offers superior renal function preservation and safety outcomes, particularly regarding major complications.

Therefore, we conducted this systematic review and meta-analysis of randomized controlled trials (RCTs) and matched cohort studies to compare off-clamp versus on-clamp RAPN with respect to perioperative and functional outcomes, including safety and efficacy outcomes. Our analysis focuses explicitly on procedures involving clamping of the main renal artery only in the robot-assisted setting.

## Review

Methods

The study’s methodology was conducted in accordance with the Cochrane Handbook for Systematic Reviews of Interventions [14]. The manuscript was prepared following the Preferred Reporting Items for Systematic Reviews and Meta-Analyses (PRISMA) guidelines [15].

Protocol Registration

This systematic review and meta-analysis were prospectively registered with the International Prospective Register of Systematic Reviews (PROSPERO; registration number: CRD42025123064).

Search Strategy

A comprehensive systematic literature search was conducted across multiple electronic databases until 2 October 2025 to identify studies examining off-clamp and on-clamp RAPN in kidney cancer. The databases included PubMed, Web of Science (WOS), Cochrane, and Scopus. The search strategy combined controlled vocabulary (Medical Subject Headings (MeSH) terms) and free-text keywords for “Renal cancer,” “on-clamp,” and “off-clamp.” Database-specific search terminology is presented in the Appendices.

Study Selection

All identified records from the database searches were imported into EndNote X9 (Clarivate Analytics, Philadelphia, PA, USA), and duplicate entries were subsequently removed. A two-phase screening methodology was employed: preliminary screening of titles and abstracts, followed by comprehensive full-text evaluation. During the initial screening phase, two independent investigators assessed all titles and abstracts according to predetermined eligibility criteria. Disagreements were adjudicated through collaborative discussion until consensus was achieved. We followed predefined inclusion criteria: studies were included if they met the following criteria.

Inclusion criteria: Studies were included if they met the following criteria: adult patients (≥18 years) diagnosed with kidney cancer, renal tumors, renal masses, or renal cell carcinoma (RCC) undergoing RAPN; intervention involving off-clamp, clampless, or zero ischemia techniques; comparison with on-clamp techniques involving main renal artery clamping or warm ischemia during RAPN; outcomes including renal functional parameters (changes in estimated glomerular filtration rate (eGFR)), perioperative measures (WIT, operative time, estimated blood loss, blood transfusion requirements, hospital stay duration), surgical safety (major complications), and oncological outcomes (positive surgical margins); and study designs comprising RCTs, non-RCTs, and observational studies with matched cohorts.

Exclusion criteria: Studies were excluded if they met any of the following criteria: case reports, case series, reviews, conference abstracts, letters, or replies; studies involving transition approaches where conversion from off-clamp to on-clamp techniques or vice versa occurred during the same procedure; studies comparing different clamping techniques such as selective arterial clamping versus complete hilar clamping; procedures performed via laparoscopic, open, or non-robotic approaches; publications in non-English languages; studies with overlapping or duplicate patient populations; or studies lacking sufficient data for meta-analysis extraction.

Data Extraction

Independent data extraction was performed by two reviewers using a standardized, pre-piloted extraction form. Discrepancies between reviewers were resolved through discussion and consensus, with consultation of a third reviewer when necessary. Extracted data included study characteristics (study ID, first author, publication year, country, study design, study period, sample size, number of centers, data collection method, inclusion and exclusion criteria, primary endpoints, and study conclusions); participant demographics (mean age ± standard deviation or median with interquartile range, sex distribution, body mass index, American Society of Anesthesiologists score ≥3, race, smoking status); comorbidities (diabetes mellitus, hypertension, cardiovascular disease); baseline renal function (preoperative serum creatinine, preoperative estimated glomerular filtration rate, CKD stage ≥3); tumor characteristics (clinical tumor size, clinical T stage, RENAL nephrometry score, PADUA score, tumor location); surgical details (robotic system used, surgical approach, number of surgeons, surgeon experience, clamping method in on-clamp group, hilar preparation in off-clamp group, use of intraoperative ultrasound, hemostatic agents); and outcome measures. Primary outcome data extracted included changes in eGFR, WIT (for the on-clamp group), operative time, estimated blood loss, blood transfusion requirements, positive surgical margins, major complications, and hospital stay duration. For each outcome, quantitative data were collected for both off-clamp and on-clamp groups, including event counts, total sample sizes, means ± standard deviations, or medians with interquartile ranges, and 95% confidence intervals where available.

Risk of Bias Assessment

The risk of bias for each study in the review was assessed by two independent reviewers using the Newcastle-Ottawa Scale (NOS), which is specifically designed to evaluate observational studies [16]. The NOS evaluates study quality across the following three domains: selection of study groups, comparability of groups, and ascertainment of exposure or outcome. Each study was systematically assessed for potential sources of bias, including participant selection criteria, adequacy of follow-up, outcome measurement methods, and control for confounding variables. Any disagreements between reviewers regarding bias assessment were resolved through collaborative discussion or consultation with a third reviewer when consensus could not be reached. The overall risk of bias for each study was categorized as poor, fair, or good based on the NOS scoring system. For RCTs included in the review, we utilized the Cochrane Risk of Bias 2 (ROB2) tool [17], which assesses bias across five specific domains: bias arising from the randomization process, bias due to deviations from intended interventions, bias due to missing outcome data, bias in measurement of the outcome, and bias in selection of the reported result. Each domain was rated as low risk, some concerns, or high risk of bias. Similar to the NOS assessment, two independent reviewers evaluated each RCT, with discrepancies resolved through discussion or third-party arbitration.

Statistical Analysis

All statistical analyses were conducted using R software version 4.4.1 (R Foundation for Statistical Computing, Vienna, Austria) [18]. Statistical significance was defined as a p-value less than 0.05. Dichotomous outcomes were analyzed using risk ratios (RRs) with 95% confidence intervals (CIs), and continuous variables were assessed using mean differences (MDs) with 95% CIs. The degree of heterogeneity across studies was evaluated using the I^2^ statistic in conjunction with the chi-square test. Significant heterogeneity was identified when the chi-square test yielded a p-value < 0.1 or when I^2^ exceeded 50%. When data were homogeneous, a fixed-effect model was used; when substantial heterogeneity was present among the included studies, a random-effects model was used.

Subgroup and Sensitivity Analyses

To explore sources of heterogeneity, prespecified subgroup analyses were performed for the primary outcome (glomerular filtration rate change) based on: (1) study design (RCTs versus observational studies), and (2) follow-up duration (<3 months, 3-9 months, and ≥12 months). Sensitivity analyses were conducted to assess the robustness of findings by systematically excluding studies with a high risk of bias or poor methodological quality.

Publication Bias Assessment

Publication bias was assessed for all outcomes using funnel plot visual inspection and Egger's regression test for funnel plot asymmetry. A p-value <0.05 in Egger's test was considered indicative of significant small-study effects or publication bias. Funnel plots for all outcomes are presented in the Results section.

Results

Study Selection

**Figure 1:**
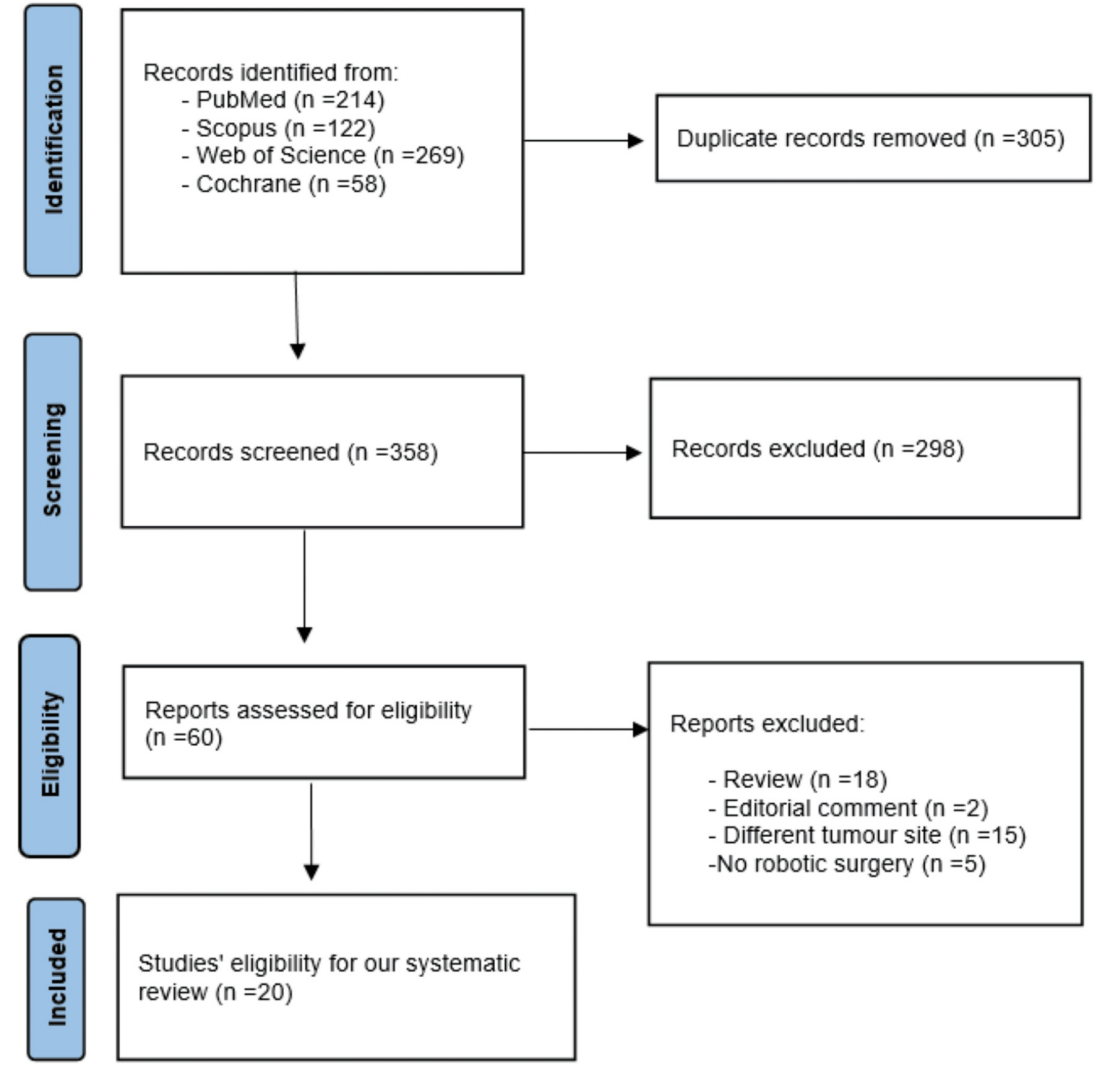
PRISMA Flow Diagram PRISMA: Preferred Reporting Items for Systematic Reviews and Meta-Analyses

After searching four electronic databases (PubMed, Scopus, WOS, and Cochrane), we identified 663 records (PubMed: n = 214; Scopus: n = 122; WOS: n = 269; Cochrane: n = 58). After removing 305 duplicates, 358 unique records remained for screening. Title and abstract screening led to the exclusion of 298 records that did not meet our eligibility criteria. We retrieved the full text of the remaining 60 articles and assessed them for eligibility. During full-text evaluation, 40 studies were excluded for the following reasons: review articles (n = 18), editorial comments (n = 2), inappropriate tumor site (n = 15), and non-robotic approach (n = 5). Finally, 20 studies were included in the systematic review and meta-analysis [19-38]. The PRISMA flow diagram illustrating the study selection process is presented in Figure 1.

Characteristics of the Included Studies

This systematic review included 20 studies, comprising a diverse range of designs: two RCTs and 18 observational studies. The total initial cohort across all studies included 10,152 participants before matching, and the final matched or analyzed cohort totaled 4,961 participants after matching. The studies were conducted in multiple countries, with the majority from the United States (n = 9), followed by Italy (n = 5), and multi-national collaborations (n = 3). Additional studies originated from Turkey (n = 2), France (n = 2), Belgium (n = 1), and China (n = 1). The publication years ranged from 2012 to 2024, with the majority (n = 15, 75%) published between 2018 and 2024, reflecting recent advancements in robotic surgical techniques. The mean age of participants ranged from 54.0 to 64.0 years across studies, with a predominantly male population. Body mass index varied considerably, from 24.0 to 32.4 kg/m^2^, encompassing normal weight, overweight, and obese patients. Baseline renal function was generally preserved, with preoperative eGFR ranging from 41.3 to 92.9 mL/min/1.73m^2^. The prevalence of CKD stage ≥3 ranged from 0% to 37.0% across studies. Comorbidities were common, with diabetes mellitus present in 5% to 21.9% of patients, hypertension in 39.4% to 65.9%, and smoking history documented in selected studies.

Tumor characteristics showed considerable variation. Clinical tumor size ranged from 1.8 to 5.5 cm, with most studies including T1a and T1b tumors. Tumor complexity was assessed using the RENAL nephrometry score (ranging from 5.3 to 8.9) or PADUA score, where reported. The majority of studies (n = 12) included predominantly cT1a tumors, while three studies specifically focused on larger tumors (cT1b-cT2). Tumor location varied, with a relatively balanced distribution between the right and left sides and involvement of the upper, mid, and lower poles. The surgical approach was predominantly transperitoneal (utilized in 15 studies either exclusively or as the primary approach), with the retroperitoneal approach used in five studies. Most studies (n = 14) reported the use of intraoperative ultrasound for tumor identification and margin delineation. Follow-up duration varied from 90 days to three years postoperatively, with most studies providing intermediate-term follow-up data. Detailed baseline characteristics and study summaries are presented in Tables 1-2.

**Table 1:** Baseline Characteristics of the Included Studies ASA: American Society of Anesthesiologists score, BMI: body mass index, CA: cancer/carcinoma, CKD: chronic kidney disease, CT2: clinical tumor stage 2, DM: diabetes mellitus, ERASE: endoscopic robot-assisted simple enucleation, HTN: hypertension, ID: identification, IQR: interquartile range, MAC: main arterial clamping, NA: not available/not applicable, OH: Ohio, PADUA: preoperative aspects and dimensions used for an anatomical nephrometry score, RAPN: robot-assisted partial nephrectomy, RENAL: radius, exophytic/endophytic, nearness, anterior/posterior, location nephrometry score, RPN: robotic partial nephrectomy, RSF: surgeon initials for Robert Sherburne Figenshau, SD: standard deviation, WIT: warm ischemia time

N	Study ID	Study arms, n (%)	Demographics (Mean ± SD or Median (IQR))					Comorbidities, n (%)				Baseline Renal Function			Tumor Characteristics		Complexity Scores, n (%)		Tumor Location, n (%)							Robotic System & Approach				Technique Details			
			Age,(mean ±SD) y	Male, n (%)	BMI (kg/m^2^) (mean ±SD)	ASA Score ≥3, n (%)	Race (White individual), n (%)	Smoking (%)	DM (%)	HTN (%)	Cardiovascular disease	Preop creatinine (mg/dL)	Preop eGFR (mL/min/1.73m^2^)	CKD Stage ≥3, n(%)	Clinical tumor size (cm)	Clinical T stage: cT1a n(%)- cT1b n(%)- cT2a n(%)- cT2b n(%)	RENAL nephrometry score	PADUA score	Right side	Left side	Upper pole	Mid pole	Lower pole	Anterior	Posterior	Robotic system: da Vinci Si/Xi/X/S/Other:____	Surgical approach: Transperitoneal/Retroperitoneal/Both	Number of surgeons: (N)	Surgeon experience: (cases/year or total cases)	Clamping method (on-clamp group): Main artery/Selective/Super-selective	Hilar preparation in off-clamp: Yes/No	Use of intraoperative ultrasound: Yes/No	Hemostatic agents used: (specify)
1	Brassetti et al.(2022) [35]	On-Clamp (89)	59.66 ± 10.54	63 (71%)	26.9 ± 4.89	37 (42%)	NA	NA	NA	NA	NA	NA	77.00 ± 23.41	NA	8	cT2 100	≥10: 36%	NA	NA	NA	NA	NA	NA	NA	NA	NA	transperitoneal	Multiple surgeons across 17 institutions		mixed: Complete hilum clamping (main renal artery + vein) and Artery-only control (selective clamping) Complete hilum clamping (main renal artery + vein)	Not specified	NOT SPECIFIED	Mixed: Renorrhaphy AND sutureless techniques
		off-clamp (89)	58.33 ± 14.31	50 (56%)	26.00 ± 4.30	29 (33%)	NA	NA	NA	NA	NA	NA	80.80 ± 21.56	NA	8	cT2 100	≥10: 43%	NA	NA	NA	NA	NA	NA	NA	NA	NA							
2	Mari et al. (2018) [22]	On-clamp ERASE (120)	61.5 ± 11.9	72 (60.0%)	25.5 ± 5.40	14 (11.7%)	NA	NA	8.3	NA	Hypertensive cardiomyopathy, n (%): 16 (13.3%), History of myocardial infarction, n (%): 17 (14.2%)	0.70 ± 0.07	90.46 ± 22.73	11 (9.2%)	2.8±1.05	cT1a n(%) 107 (89.2%)- cT1b n(%) 13 (10.8%) - cT2a n(%) 0 (0%)- cT2b n(%) 0 (0%)	NA	6-7: 85 (73.3%), 8-9: 31 (26.7%), ≥10: 0 (0%)	57 (47.5%)	63 (52.5%)	44 (36.7%)	53 (44.2%)	23 (19.2%)	NA	NA	Da Vinci 4S	transperitoneal	3 surgeons	Highly experienced; first 25 cases per surgeon (learning curve) excluded	Complete hilum clamping			Standardized double-layer running technique: 1st layer: Medulla (2-0 monofilament, 26mm needle) 2nd layer: Cortical (2-0 polyglactin 910, 31mm needle)
		Off-clamp ERASE (120)	62.2 ± 12.2	73 (60.8%)	25.9 ± 6.15	15 (12.5%)	NA	NA	5	NA	Hypertensive cardiomyopathy N(%): 10 (8.3%), History of myocardial infarction, n (%): 19 (15.8%)	0.84 ± 0.18	87.7 ± 24.91	13 (10.8%)	2.46±1.27	cT1a n(%) 108 (90.0%) - cT1b n(%) 12 (10.0%) - cT2a n(%) 0 (0%)- cT2b n(%) 0 (0%)	NA	6-7: 90 (76.3%), 8-9: 28 (23.7%), ≥10: 0 (0%)	54 (45.0%)	66 (55.0%)	35 (29.2%)	58 (48.3%)	27 (22.5%)	NA	NA	Da Vinci 4S		3 surgeons			YES		
3	Antonelli et al. (2022) [32]	On-clamp RAPN (160)	62.3 ± 11.9	86 (60%)	26.5 ± 3	NA	NA	NA	21 (13%)	85 (53%)	Vascular disease, n (%): 28 (17%), Cardiac disease, n (%): 40(25%	NA	86.3 ± 15.4	0%	3±1.3	NA	6.66±2.24, RENAL score ≤10: (100%)	NA	NA	NA	NA	NA	NA	NA	NA	NA	transperitoneal	7 surgeons (one per institution)	Well-defined profile: Age <45 years; Previous experience ≥100 RAPNs; Experience with both on- and off-clamp approaches	Global ischemia until completion of medullary renorrhaphy	Yes, mandatory in both arms		NA
		Off-clamp RAPN (164)	64 ± 11.9	99 (60%)	26.2 ± 3.1	NA	NA	NA	17 (10%)	95 (58%)	Vascular disease, n (%): 25 (15%), Cardiac disease, n (%): 29 (18%)	NA	87.2 ± 16.6	0%	2.8±1.34	NA	6±1.4, RENAL score ≤10: (100%)	NA	NA	NA	NA	NA	NA	NA	NA	NA							
4	Anderson et al. (2018) [20]	On-clamp RAPN (50)	56.9 ± 13.4	32 (64.0%)	31.0 ± 7.6	NA	NA	NA	NA	NA	NA	NA	86.4 ± 27.5	NA	3.5 ± 1.5	NA	7.8 ± 1.7	NA	23 (46.0%)	27 (54.0%)	NA	NA	NA	NA	NA	12-mm camera trocar, three 8-mm robotic trocars	Transperitoneal (flank position with table flexed)	Single surgeon (RSF)	>300 off-clamp RAPNs performed; "extensive experience in robotic surgery"	Traditional hilar clamping with bulldog clamps	YES, typically dissect the hilar vessels to facilitate bulldog clamp application in case excessive bleeding is encountered	YES, typically use intraoperative ultrasound to help demarcate tumor margins and depth	Surgicel (Ethicon, Cincinnati, OH); Deliberate electrocautery for bleeding vessels; ProGrasp forceps for direct pressure, Renorrhaphy technique: Standardized: 1. Inner-layer: 2-0 V-Loc (Medtronic) 2. Outer-layer: Sliding-clip renorrhaphy with interrupted 0 Vicryl + Hem-o-lok clips + Lapra-Ty suture clips
		Off-clamp RAPN (50)	59.4 ± 11.4	22 (44.0%)	31.2 ± 6.8	NA	NA	NA	NA	NA	NA	NA	81.5 ± 28.3	NA	3.4 ± 1.7	NA	7.5 ± 2.1	NA	25 (50.0%)	25 (50.0%)	NA	NA	NA	NA	NA								
5	Anderson et al. (2019) [31]	On-clamp RAPN (40)	59.4 ± 11.2	29 (72.5%)	31.6 ± 5.9	NA	NA	NA	NA	NA	NA	NA	92.0 ± 21.6	NA	3.1 ± 1.4	cT1 only: NA, CT2: 0%	7.3 ± 2.0	NA	24 (60.0%)	16 (40.0%)	NA	NA	NA	NA	NA	DaVinci Si System (Intuitive Surgical Inc., Sunnyvale, CA), 5 trocars: Two 12-mm (camera + assistant), three 8-mm robotic (4-arm setup)	Transperitoneal (standard flexed lateral decubitus position)	Single surgeon (RSF)	>300 off-clamp RAPNs; "extensive experience in robotic surgery"	Bulldog or second ProGrasp on main renal artery; No intravenous mannitol	yes, Dissection of kidney and hilar vessels performed similarly in both groups	YES - Used to delineate tumor margins in both groups	ProGrasp forceps pressure; Hemostatic agents (Surgicel); Clamp main renal artery if excessive bleeding, Resection technique (off-clamp): Electrocautery circumferentially around mass; Gentle lifting of mass; Precise retraction + electrosurgical energy, Resection technique (on-clamp): Sharp excision after clamping, Renorrhaphy: Standardized: Inner layer: 2-0 V-Loc (corticomedullary) Outer layer: Sliding-clip technique with 0 Vicryl + Hem-o-lok + Lapra-Ty clips
		Off-clamp RAPN (40)	56.6 ± 9.8	22 (55.0%)	32.4 ± 6.3	NA	NA	NA	NA	NA	NA	NA	85.8 ± 21.0	NA	2.9 ± 1.2	cT1 only: NA, CT2: 0%	6.8 ± 2.0	NA	18 (45.0%)	22 (55.0%)	NA	NA	NA	NA	NA								
6	Kaczmarek et al. (2013) [21]	OFF-clamp RPN (49)	60.4 ± 1.5	24 (49.0%)	29.7 ± 0.9	5 (10.2%)	Caucasian: 39 (79.6%), African American: 8 (16.3%), Other: 2 (4.1%)	NA	NA	NA	NA	NA	85.2 ± 3.0	NA	2.9 ± 0.2	NA	5.3 ± 0.2	NA	NA	NA	NA	NA	NA	NA	NA	da Vinci Surgical System (Intuitive Surgical Inc., Sunnyvale, CA)	NOT SPECIFIED	5 surgeons (one per institution; all high-volume, experienced)	High-volume surgeons with experience in both on- and off-clamp approaches	Transient hilar clamping to maintain visualization during tumor excision and reconstruction	YES - "The renal artery was routinely localized and dissected in case hilar clamping became necessary"	YES, After the Gerota fascia was opened, the tumor was identified and demarcated with the assistance of intraoperative ultrasonography	HEMostatic agents used: NA, renorraphy: Inner parenchymal layer sutured reconstruction, followed by capsular sutures
		on- clamp RPN (controls) (283)	60.2 ± 0.5	122 (43.1%)	29.9 ± 0.3	15 (5.3%)	Caucasian: 229 (80.9%), African American: 41 (14.5%), Other: 13 (4.6%)	NA	NA	NA	NA	NA	82.5 ± 1.3	NA	2.9 ± 0.1	NA	5.6 ± 0.1	NA	NA	NA	NA	NA	NA	NA	NA								
7	Rosen et al. (2017) [26]	Off-Clamp (Off-C) (41)	61 ± 14.5	26 (63.4%)	28.7 ± 4.37	28 (68.3%)	NA	NA	9 (21.9%)	27 (65.9%)	3 (7.3%) [Coronary Artery Disease]	NA	79.8 ± 27.5	7 (17.1%) [baseline CKD]	1.8±0.7	T1a (all patients), cT1a n(%):100%	5.3±2.3	NA	11 (26.8%)	30 (73.2%)	9 (22.5%)	18 (45.0%)	13 (32.5%)	19 (46.3%)	17 (41.5%)	Da Vinci Surgical System (not specified)	Transperitoneal	5	Off-C utilized at rates of 32% (n=12), 20% (n=34), 15% (n=11), 0% (n=0), 0% (n=0) among the 5 surgeons	Main artery	Yes (renal artery dissected and identified with vessel loop for rapid hilar control if needed)	NA	NA
		Main Arterial Clamping (MAC) (82)	61 ± 13.9	47 (57.3%)	30.1 ± 5.8	53 (64.6%)	NA	NA	13 (15.9%)	51 (62.2%)	7 (8.5%) [Coronary Artery Disease]	NA	82.9 ± 25.5	11 (13.4%) [baseline CKD]	2±0.6		5.3±1.5	NA	26 (31.7%)	56 (68.3%)	24 (35.8%)	21 (31.3%)	22 (32.8%)	35 (42.7%)	37 (45.1%)								
8	Güner et al. (2021) [37]	On-clamp (n=78)	59.4 ± 11.2	49 ± 62.8	NA	88 ± 62.0	NA	NA	15 ± 19.2	38 ± 48.7	NA	NA	77.2 ± 20.7	NA	3.5 ± 1.85	NA	7.00 ± 2.22	NA	745 ± 39.12	1159 ± 60.88	600 ± 31.5	735 ± 38.6	569 ± 29.9	958 ± 50.3	946 ± 49.7	Da Vinci Surgical System (not specified)	Transperitoneal 1628 (85.5) and Retroperitoneal 276 (14.5)	NA	Patients on learning curve excluded	Renal artery clamped with bulldogs	Not specified	Yes (to identify renal mass)	2-0 Vicryl for vessel hemostasis/collecting system, 3-0 V-Loc for parenchymal hemostasis
		Off-clamp (n=78)	59.2 ± 15.6	52 (66.7%)	NA	43 ± 20.3	NA	NA	11 ± 14.1	35 ± 44.9	NA	NA	80.5 ± 22.6	NA	3.1 ± 2	NA	6.00 ± 2.22	NA	95 ± 45.2	115 ± 54.8	72 ± 34.2	75 ± 35.8	63 ± 30	128 ± 61	82 ± 39		Transperitoneal 186 (88.5) and Retroperitoneal 24 (11.4)			NA	Renal hilum dissected, vessel loop used		
9	Vargo et al. (2024) [30]	On-clamp (n=65)	62.00 ± 13.70	39 ± 60.0	27.40 ± 6.59	32 ± 49.2	NA	NA	NA	NA	NA	1.03 ± 0.57	77.2 ± 20.7	NA	5.1 ± 1.1	Study included cT1b-cT2N0M0	8.9 ± 1.4	NA	NA	NA	NA	NA	NA	NA	NA	Da Vinci Surgical System (Intuitive Surgical, Sunnyvale, CA)	NOT SPECIFIED	NA	NA	Renal artery clamped with bulldogs	Not specified	Yes (renal mass identified)	Two-layer renorrhaphy performed
		Off-clamp (n=65)	61.00 ± 12.59	43 ± 66.2	27.50 ± 5.48	34 ± 52.3	NA	NA	NA	NA	NA	0.97 ± 0.31	78.66 ± 25.8	NA	5.5 ± 1.7	Study included cT1b-cT2N0M0	8.7 ± 1.9	NA	NA	NA	NA	NA	NA	NA	NA		NOT SPECIFIED	NA	NA	NA	Renal hilum dissected completely		
10	Tuderti et al. (2023) [29]	On-clamp (n=78)	61.00 ± 12.59	55 ± 53.6	27.50 ± 5.48	30 ± 35.9	NA	NA	NA	NA	NA	0.97 ± 0.31	78.66 ± 25.8	NA	4.92 ± 3.45	Study focused on cT1-cT2 with RENAL score ≥9	score 9:44 (31), score 10:78 (54.9), score 11: 19 (13.4), score 12: 1 (0.7)	NA	NA	NA	NA	NA	NA	NA	NA	Da Vinci Surgical System (not specified)	NOT SPECIFIED	Multiple surgeons	High experience	Renal artery clamped with bulldogs	Not specified	Yes (to identify renal mass and mark borders)	Two-layer renorrhaphy after excision
		Off-clamp (n=78)	62.00 ± 13.70	63 ± 60	27.40 ± 6.59	34 ± 41	NA	NA	NA	NA	NA	1.03 ± 0.57	75.6 ± 25.5	NA	5.29 ± 2.47 cm	Study focused on cT1-cT2 with RENAL score ≥9	score 9:73 (34.4), score 10: 101 (47.6), score 11: 29 (13.7), score 9: 1 (4.2)	NA	NA	NA	NA	NA	NA	NA	NA		NOT SPECIFIED	Multiple surgeons	High experience	NA	Renal hilum dissected completely		
11	Sharma et al. (2023) [27]	On-clamp (n=205)	61.00 ± 12.59	110 ± 53.6	27.50 ± 5.48	2 ± 0.97	NA	NA	NA	NA	NA	0.97 ± 0.31	78.66 ± 25.8	NA	3.34 ± 1.59	NA	7.00 ± 2.22	NA	76 ± 37.1	129 ± 62.9	58 ± 28.3	75 ± 36.5	72 ± 35.1	107 ± 52.2	98 ± 47.8	Da Vinci Surgical System (Intuitive Surgical)	Transperitoneal: 174 (84.8) Retroperitoneal: 31 (15.2)	Multiple	High experience	NA	NA	NA	NA
		Off-clamp (n=205)	62.00 ± 13.70	123 ± 60	27.40 ± 6.59	2 ± 0.97	NA	NA	NA	NA	NA	1.03 ± 0.57	75.6 ± 25.5	NA	3.1 ± 2	NA	6.00 ± 2.22	NA	93 ± 45.3	112 ± 54.7	70 ± 34.1	73 ± 35.6	62 ± 30.2	125 ± 61	80 ± 39	Da Vinci Surgical System (Intuitive Surgical)		Multiple	High experience	NA	NA	NA	NA
12	Rac et al. (2024) [25]	On-clamp: 124	57.8 ± 12.5	79 (63.7%)	31.8 ± 6.1	NA	86 (69.4%)	NA	NA	NA	NA	NA	81.2 ± 23.5	NA	3.0 ± 1.1	NA	6.0 ± 1.6	7.8 ± 1.4	64 (51.6%)	60 (48.4%)	NA	NA	NA	NA	NA	da Vinci (system not specified)	Transperitoneal: 26 (21.0%); Retroperitoneal: 98 (79.0%)	1	NA	Hilar clamping with bulldog clamps	NA	NOT SPECIFIED	TachoSil, Hem-O-Locs, barbed sutures
		Off-clamp: 65	57.9 ± 13.4	42 (64.6%)	30.8 ± 7.8	NA	52 (80.0%)	NA	NA	NA	NA	NA	76.5 ± 22.1	NA	2.5 ± 1.2	NA	6.0 ± 1.5	7.2 ± 1.0	36 (55.4%)	29 (44.6%)	NA	NA	NA	NA	NA	da Vinci (system not specified)	Transperitoneal: 26 (40.0%); Retroperitoneal: 39 (60.0%)	1	NA	NA	Yes (renal artery dissected with vessel loop)	NOT SPECIFIED	TachoSil, Hem-O-Locs, barbed sutures
13	Guo et al. (2019) [38]	On-clamp: 45	54.39 ± 11.82	25 (55.6%)	24.03 ± 4.21	NA	NA	NA	NA	NA	NA	0.93 ± 0.2	42.84 ± 5.03	NA	3.50 ± 0.76	NA	8.6 ± 1.8	NA	21 (46.7%)	24 (53.3%)	11 (24.4%)	20 (44.4%)	14 (31.1%)	NA	NA	da Vinci Si	Retroperitoneal: 45 (100%)	1	NA	Main renal artery with bulldog clamp	NA	Utilized	TachoSil, barbed sutures, Hem-O-Locs
		Off-clamp: 48	53.29 ± 13.91	27 (56.3%)	24.63 ± 3.44	NA	NA	NA	NA	NA	NA	0.84 ± 0.6	41.25 ± 4.79	NA	3.28 ± 0.64	NA	8.4 ± 1.7	NA	22 (45.8%)	26 (54.2%)	9 (18.8%)	25 (52.1%)	14 (29.2%)	NA	NA	da Vinci Si	Retroperitoneal: 48 (100%)	1	NA	NA	Yes (renal artery dissected, bulldog clamp ready)	Utilized	TachoSil, barbed sutures, Hem-O-Locs
14	Ener et al. (2016) [36]	On-clamp: 22	54.4 ± 10.1	19 (86.4%)	28.3 ± 3.5	NA	NA	NA	NA	NA	NA	0.93 ± 0.2	83.9 ± 21.5	NA	3.2 ± 0.9	NA	6.0 ± 1.6	7.8 ± 1.4	NA	NA	NA	NA	NA	NA	NA	da Vinci Si	Transperitoneal: 22 (100%)	1 (single surgical team across 2 centers)	NA	Laparoscopic bulldog clamp on renal artery	NA	Not specified	TachoSil, barbed sutures, Lapra-Ty clips
		Off-clamp: 12	53.0 ± 8.2	11 (91.7%)	29.2 ± 4.6	NA	NA	NA	NA	NA	NA	0.84 ± 0.6	92.9 ± 29.8	NA	3.3 ± 1.1	NA	6.0 ± 1.5	7.2 ± 1.0	NA	NA	NA	NA	NA	NA	NA	da Vinci Si	Transperitoneal: 12 (100%)	1 (single surgical team across 2 centers)	NA	NA	Yes (renal artery dissected with vessel loop)	Not specified	TachoSil, barbed sutures, Lapra-Ty clips
15	Belmonte et al. (2024) [33]	On-clamp (n=224)	62.50 ± 2.37	64.8	27	NA	NA	NA	NA	NA	NA	0.91 ± 0.12	69.90 ± 4.07	NA	3.90 ± 0.44	Similar distribution after weighting	NA	8.98 ± 1.85	55.80%	43.50%	NA	NA	NA	NA	NA	da Vinci Si, Xi, X	Transperitoneal: 95% and Retroperitoneal: 5%	4 expert urologists	NA	Main renal artery clamping	NA	NA	NA
		Off-clamp (n=86)	61.70 ± 7.85	64.50%	28.3	NA	NA	NA	NA	NA	NA	1.21 ± 0.61	71.10 ± 6.81	NA	4.20 ± 1.04		NA	8.27 ± 0.37	66.90%	33.10%	NA	NA	NA	NA	NA	da Vinci Si, Xi, X	Transperitoneal: 99.1% and Retroperitoneal: 0.9%		NA	NA	NA	NA	NA
16	Mutelica et al. (2020) [23]	On-clamp (n=309)	60.00 ± 11.11	174 (58.0%)	210 (68.0%)	210 (68.0%)	264 (85.4%)	NA	NA	NA	NA	NA	84.00 ± 21.48	113 (36.5%)	2.00 ± 0.74	pT1a: 230 (74.4%) vs 69 (67%); pT3a: 17 (5.5%) vs 4 (3.9%) (p=0.8)	<7: 216 (69.9%), ≥7: 93 (30.1%)	NA	160 (51.8%)	149(48.2%)	NA	NA	NA	NA	NA	da Vinci Surgical System (Si, Xi, X)	NOT SPECIFIED	8 senior surgeons	ranging from 20 to 50 procedures	Main renal artery; early unclamping in 54.8% (n=57/104 on-clamp patients)	37 (12%)	NA	NA
		Off-clamp (n=103)	63.00 ± 11.85	57 (55.3%)	75 (72.8%)	75 (72.8%)	86(83.5%)	NA	NA	NA	NA	NA	83.00 ± 22.22	24 (23.3%)	2.00 ± 0.81		<7: 78 (75.7%) ≥7: 25 (24.3%)	NA	44 (42.7%)	59(57.3%)	NA	NA	NA	NA	NA	da Vinci Surgical System (Si, Xi, X)	NOT SPECIFIED			NA	10 (9.7%)	NA	NA
17	Peyronnet et al. (2017) [24]	On-clamp (n=104)	63.8	NA	26.4	12 (11.5%)	NA	NA	NA	NA	NA	NA	78.4	NA	25.3	T1a: 79 (76.1%) vs 21 (80.2%)		NA	NA	NA	NA	NA	NA	NA	NA	da Vinci (not specified)	Both transperitoneal and retroperitoneal approaches included	Not specified from 8 inistitutions	more than 50 cases	Main renal artery ± early unclamping	Not specified	NA	NA
		Off-clamp (n=26)	59.7	NA	26.1	4 (15.5%)	NA	NA	NA	NA	NA	NA	84.9	NA	26.4			NA	NA	NA	NA	NA	NA	NA	NA	da Vinci (not specified)	Both transperitoneal and retroperitoneal approaches included			NA	yes included	NA	NA
18	Bertolo et al. (2019) [34]	On-clamp RAPN (400)	59.2 ± 12.1	269 (67.2%)	31.0 ± 7.6	146 (36.5%)	NA	107 (26.7%)	83 (20.7%)	210 (52.5%)	NA	NA	81.9±24.0	NA	4.0±2.7	NA	7.5±2.0	NA	98 (47.8%) combined	98 (47.8%) combined	NA	NA	NA	NA	NA	Not specified	Transperitoneal (primary)	Multiple surgeons across 2 institutions	Experienced; >300 off-clamp RAPNs performed (RSF)	Mixed: Main artery + vein (complete hilum)/Selective artery-only	Not specified	Yes - to demarcate tumor margins and depth	Surgicel (Ethicon); Electrocautery; ProGrasp forceps pressure. Renorrhaphy: 2-0 V-Loc inner layer, 0 Vicryl + Hem-o-lok + Lapra-Ty clips outer layer
		Off-clamp RAPN (200)	60.2 ± 11.6	146 (73%)	31.2 ± 6.8	68 (34%)		45 (22.5%)	37 (18.5%)	101 (50.5%)	NA	NA	81.1±21.4	NA	4.2±2.8	NA	7.7±1.9	NA	107 (52.2%) combined	107 (52.2%) combined	NA	NA	NA	NA	NA	Not specified				NA	hilar vessels dissected for potential emergency clamping		
19	AnCeSChi et al. (2022) [19]	On-clamp RAPN (104)	78 (76-80)	65 (62.5%)	25.1 (23.4-27.7)	62 (59.6%)	NA	NA	11 (10.6%)	41 (39.4%)	NA	0.84±0.18	66.9 (52.8-80.3)	37 (35.6%)	3.0 (2.2-4.1)	cT1a: 79 (76%); cT1b: 22 (21.2%); cT2a: 3 (2.9%); cT2b: 0	NA	NA	56 (53.8%)	48 (46.2%)	NA	NA	NA	NA	NA	Da Vinci	Transperitoneal: 91 (87.5%); Retroperitoneal: 13 (12.5%)	7 surgeons (one per institution; 7 Italian centers)	Age <45 yrs; ≥100 RAPNs; experienced in both on/off-clamp	Main artery + vein (global ischemia); Selective in 46 pts (44.2%) with median WIT 15 min (10-19.2)	Not specified	Yes - to assess tumor depth and delineate resection site	On-clamp: Cold scissors + mannitol. Off-clamp: Electrocautery 50W (79.3%) or Habib-4X device (20.7%); no mannitol. Both: Monopolar/bipolar cautery for vessels; 2.0-Vicryl for collecting system; 0-Vicryl mattress + Weck clips + Lapra-Ty
		Off-clamp RAPN (101)	78 (76-80)	68 (67.3%)	25.7 (24-27.2)	65 (64.4%)	NA	NA	9 (8.9%)	50 (49.5%)	NA	0.70±0.07	66.1 (60.7-87)	24 (23.8%)	3.8 (2.5-4.8)	cT1a: 60 (59.4%); cT1b: 38 (37.6%); cT2a: 3 (3%); cT2b: 0	NA	NA	42 (41.6%)	59 (58.4%)	NA	NA	NA	NA	NA	Da Vinci	Transperitoneal: 18 (17.8%); Retroperitoneal: 83 (82.2%)				Yes - mandatory; hilar dissection for emergency control if excessive bleeding		
20	Tanagho et al. (2012) [28]	On-clamp RAPN (29)	60.0±13.4	NA	29.0±7.9	NA	NA	NA	3/24 (12.5%)	13/24 (54.2%)	NA	1.2±1.6	85.8±21.3	NA	2.3±1.4	NA	5.7±1.9	NA	NA	NA	NA	NA	NA	NA	NA	Da Vinci Si	Transperitoneal	1	>300 off-clamp RAPNs; extensive robotic experience	Main artery (bulldog clamp or ProGrasp); No mannitol	Not specified	Yes - to delineate tumor margins	Surgicel; ProGrasp forceps; Main artery clamped if excessive bleeding in off-clamp. Resection: On-clamp (cold scissors), Off-clamp (electrocautery). Renorrhaphy: 2-0 V-Loc inner, 0 Vicryl + Hem-o-lok + Lapra-Ty outer
		Off-clamp RAPN(29)	60.8±12.1	NA	30.2±7.1	NA	NA	NA	2/24 (8.3%)	14/24 (58.3%)	NA	0.9±0.2	84.8±26.7	NA	2.3±1.2	NA	5.7±1.9	NA	NA	NA	NA	NA	NA	NA	NA	Da Vinci Si	Transperitoneal	1		NA	Yes - hilar vessels dissected similarly in both groups		

**Table 2:** Summary of the Included Studies AKI: acute kidney injury, ASA: American Society of Anesthesiologists score, AV: arteriovenous, BMI: body mass index, CCI: Charlson comorbidity index, CKD: chronic kidney disease, CT: computed tomography, EB: estimated blood loss, EBL: estimated blood loss, EPI: Chronic Kidney Disease Epidemiology Collaboration (CKD-EPI), ERASE: endoscopic robot-assisted simple enucleation, GFR: glomerular filtration rate, HR: hazard ratio, ID: identification, IPTW: inverse probability of treatment weighting, IQR: interquartile range, ITT: intention-to-treat, LOS: length of stay, MAC: main arterial clamping, MRI: magnetic resonance imaging, OR: operating room or odds ratio, PADUA: preoperative aspects and dimensions used for an anatomical nephrometry score, PN: partial nephrectomy, POD1: postoperative day 1, PP: per-protocol, PSM: propensity score matching, RAPN: robot-assisted partial nephrectomy, RCT: randomized controlled trial, RENAL: radius, exophytic/endophytic, nearness, anterior/posterior, location nephrometry score, RNS: RENAL nephrometry score, RPN: robotic partial nephrectomy, RRLPN: robot-assisted retroperitoneal laparoscopic partial nephrectomy, RSF: Surgeon initials for Robert Sherburne Figenshau, SM: surgical margins, UK: United Kingdom, USA: United States of America, VCQI: Vattikuti Collective Quality Initiative, WIT: warm ischemia time

N	Study ID	Year	Study arms	Country	Study design	Study period (months)/(start date-end date)	Sample size	Data collection: Prospective/retrospective	Number of centers: single/multi-center	Primary endpoints	Conclusion	Inclusion criteria	Exclusion criteria
1	Brassetti et al. (2022) [35]	2022	on-clamp RAPN	Multi-national (USA, Italy, Belgium, South Korea, China)	Retrospective, Propensity-Score-Matched, Multicenter Outcome Analysis	July 2007 - February 2022	316 total patients (Overall: 211 on-clamp, 105 off-clamp; PSM: 89 on-clamp, 89 off-clamp)	Retrospective	multicenter (17 participating institutions)	Trifecta achievement (negative surgical margins, no severe complications, ≤30% postoperative eGFR reduction); perioperative surgical outcomes	Off-clamp RAPN is safe and viable for cT2 renal masses, with shorter operation times and significantly improved probabilities of achieving favorable perioperative outcomes	Patients who underwent RAPN for cT2 renal masses, tumor characeristics (cT2cN0cM0 kidney tumor; Clinical tumor size >7 cm)	solitary kidney: No (13 patients [4%] had solitary kidney in overall cohort), CKD and comrbid patients NOT excluded, Bilaterl tumors yes likely excluded
			off-clamp RAPN										
2	Mari et al. (2018) [22]	2018	On-clamp ERASE	Italy	Retrospective, Matched-pair comparison	January 2011 - December 2014]	491 total patients (After exclusions: 344 patients; PSM: 120 on-clamp vs 120 off-clamp)	Prospective collection, Retrospective analysis	Single-center (tertiary referral institution)	Surgical and functional outcomes; Trifecta achievement (WIT<25 min, negative SM, no complications); eGFR preservation	Off-clamp ERASE is safe with significantly lower renal function drop in early perioperative time. Patients with comorbidity (CCI ≥2) benefit from off-clamp even long-term	Patients treated by 3 highly experienced surgeons; Underwent ERASE (endoscopic robot-assisted simple enucleation), Tumor characteristics (Clinical T1 renal tumors; Tumor size: Median 2.5-2.8 cm, Tumor complexity: PADUA score 6-9 (no patients with PADUA ≥10 in PSM cohort)	1. Learning Curve Cases: First 25 cases performed by EACH of the 3 surgeons excluded, 2-35 zero-ischemia cases excluded, 3- 3 patients excluded from functional follow-up analysis, Solitary Kidney: NOT excluded, Baseline Chronic Kidney Disease: NOT excluded All CKD stages included, Comorbid patients NOT excluded
			Off-clamp ERASE										
3	Antonelli et al. (2022) [32]	2022	On-clamp RAPN	italy	Phase 3 Randomized Controlled Trial (RCT)	September 2015 - November 2018	324 randomized (160 on-clamp, 164 off-clamp); After crossover: ITT 324 patients; PP 232 patients (137 on-clamp, 95 off-clamp)	Prospective	Multi-center (7 Italian institutions)	6-month absolute variation in eGFR (AV-GFR)	NO EVIDENCE of differences in functional outcomes between on-clamp vs off-clamp RAPN in patients with regular baseline function and two kidneys	Age criteria: 18-80 years, Tumor characteristics: cT1 renal tumor with RENAL score ≤10, Baseline renal function: eGFR ≥60 mL/min/1.73m², Other inclusion criteria: Regular coagulation profile; Two kidneys; No medical history or imaging findings of abnormalities for either kidney; Signed informed consent.	Solitary kidney, Baseline CKD, RENAL score >10: YES excluded, 29 patients excluded from 353 screened (criteria not detailed)
			Off-clamp RAPN										
4	Anderson et al. (2018) [20]	2018	On-clamp RAPN	USA	Retrospective comparison using Propensity Score Matching	January 2009 - September 2015]	239 total patients; PSM: 50 on-clamp vs 50 off-clamp	Retrospective from prospectively maintained database	Single-center	Perioperative outcomes and renal functional outcomes (eGFR changes)	Off-clamp RAPN did NOT result in improved renal functional preservation. Surprisingly, off-clamp had lower EBL, shorter operative times, and fewer complications	Tumor characteristics: Clinical T1 renal masses treated with RAPN, All patients who underwent RAPN by single surgeon (RSF) 2009-2015	Incomplete data for: age, CCI, gender, BMI, pre/post eGFR, tumor size, RENAL score, pelvicaliceal repair, operative time, EBL, margins, complications → Reduced usable cohort to 50 clamped + 83 off-clamp
			Off-clamp RAPN										
5	Anderson et al. (2019) [31]	2019	On-clamp RAPN	USA	Prospective Randomized Controlled Trial (RCT)	2013 - 2017	80 patients (40 on-clamp, 40 off-clamp); 1:1 randomization	Prospective	Single-center	Postoperative renal function (eGFR and renal scintigraphy at 3 months)	Off-clamp RAPN did NOT result in improved renal functional preservation. Similar perioperative outcomes. Surgeons may safely use either approach depending on preference and patient factors	Age criteria (18-80),Tumor characteristics (Organ-confined renal mass suspicious for malignancy, deemed amenable to RAPN), Other inclusion criteria (Karnofsky Performance Status ≥40; Able to sign consent)	Unwilling to participate excluded, Solitary kidney: NOT excluded, Bilateral/multiple tumors: not excluded,Baseline CKD: Not excluded
			OFF-clamp RAPN										
6	Kaczmarek et al. (2013) [21]	2013	On-clamp RPN	USA	Retrospective with Propensity Score Matching	May 2007 - November 2011	886 total RPNs; After exclusions: 49 off-clamp vs 283 on-clamp controls; PSM: 49 off-clamp matched	Prospectively collected, retrospectively analyzed	Multi-center (5 academic institutions)	Perioperative and functional outcomes of off-clamp RPN; Comparative effectiveness vs clamped RPN	Off-clamp RPN is safe and feasible with appropriately selected patients and adequate surgeon experience. Associated with higher EBL, shorter operative times, and smaller decrease in renal function (2% vs 6% drop in eGFR, p=0.008)	Renal tumors treated with RPN, RPNs performed by high-volume surgeons across 5 institutions	Preop eGFR <30, ASA >3, Lacking RENAL score, Missing BMI, Missing ASA, Missing center assignment, Missing postop eGFR, Missing date of last eGFR, Missing OR time, Missing EB, Missing transfusion info, Missing LOS, Solitary kidney NOT excluded (5 patients [8%] in off-clamp group had solitary kidney), Bilateral/multiple tumors EXCLUDED (1 patient with multiple tumors excluded), Baseline CKD NOT excluded (13 patients [20%] had eGFR ≤60), Other exclusion criteriaCases not using off-clamp for ALL tumors excluded
			Off-clamp RPN										
7	Rosen et al. (2017) [26]	2017	Main Arterial Clamping (MAC)	USA	Retrospective cohort	January 2008 - June 2016	351 total (before matching); 123 after propensity matching (82 MAC, 41 Off-C)	Retrospective	Multi-center (5 institutions)	Renal function outcomes (eGFR change, AKI, CKD progression)	Off-C RPN marginally increased blood loss without providing renal function benefit in two-kidney patients with T1a tumors	Tumor characteristics: T1a tumor, R.E.N.A.L. Nephrometry Score <10, two kidneys present, Baseline eGFR ≥30, normal serum creatinine levels, Complete follow-up data between 3-18 months	Solitary kidney: Yes (excluded), Bilateral/multiple tumorsYes (excluded - multiple renal masses resected, n=20), Previous renal surgeryYes (excluded - previous ipsilateral kidney surgery, n=21), Baseline CKDYes (excluded if eGFR <30, n=10), RNS >9 (n=96), mass abutting main renal artery/vein (n=78), known metastatic disease (n=1), horseshoe kidney (n=2), tumor thrombus (n=2), conversion to radical/open PN (n=3), missing clamp technique data (n=22)
			Off-clamp RPN										
8	Güner et al. (2021) [37]	2021	On-clamp RPN	Turkey	Retrospective comparative analysis	January 2008 - December 2018	90 patients total	Retrospective	Single-center	Renal function (eGFR changes), Operative time, Warm ischemia time, Estimated blood loss, Complications	Findings regarding renal function tests are inadequate to state that either robotic or open zero-ischemia PN is superior to their ischemic counterpart. Besides operative time, warm ischemia time, estimated blood loss, and excised healthy renal parenchyma must be considered while predicting long-term renal function after PN.	Preoperative normal serum creatinine levels, T1 tumors, Either on-clamp or off-clamp PN (open or robot-assisted laparoscopic approach), Follow-up of at least 18 months, Complete data available	Solitary kidney, CKD, Bilateral or multiple tumors, Patients on the learning curve
			Off-clamp RPN										
9	Vargo et al. (2024) [30]	2024	On-clamp RPN	USA	Retrospective propensity score-matched analysis	2007-2021	225 patients	Retrospective	Multi-center (3 institutions)	Safety (perioperative complications), Efficacy (trifecta achievement: negative margins, no Clavien-Dindo ≥3 complications, eGFR decline ≤30%), Renal function (eGFR at 1 year)	Off-clamp RPN is a safe and effective approach for patients with T1b or greater renal tumors. Blood transfusion rate was significantly lower at 1.5% for off-clamp RPN. Risk of major complication was 6.1% lower in off-clamp cohort. Postoperative eGFR and positive margin rates were similar between groups.	cT1b-T2N0M0 renal tumors, Tumor size ≥4 cm, Robot-assisted partial nephrectomy	Not explicitly stated in abstract
			Off-clamp RPN										
10	Tuderti et al. (2023) [29]	2023	On-clamp RPN	Multi-national (Italy and USA)	Retrospective propensity score-matched comparative analysis	2003-2021	354 patients total	Retrospective	Multi-center (3 high-volume institutions)	Perioperative outcomes, Functional outcomes (eGFR changes, trifecta achievement), Probability of retaining eGFR ≥45 ml/min over time	Found favorable trade-off between benefits and risks of off-clamp RPN, with similar perioperative outcomes and net benefit in trifecta achievement (83.3% vs 67.9%; p=0.03) and long-term renal function. Off-clamp group had significantly higher probability of retaining eGFR ≥45 ml/min over time. WIT >20 min was independently negatively associated with trifecta achievement.	cT1-cT2 localized renal tumors, RENAL score ≥9, Complete data available	Bilateral or multiple renal masses (imperative RPN), Incomplete data on type of ischemia
			Off-clamp RPN										
11	Sharma et al. (2023) [27]	2023	On-clamp RPN	Multi-national (9 countries: USA, UK, India, Italy, Portugal, Belgium, Turkey, South Korea)	Retrospective propensity score-matched analysis of prospective database	October 2014 - March 2020	2114 patients total	Prospective database with retrospective analysis	Multi-center (18 institutions from 9 countries - VCQI database)	Perioperative outcomes, Functional outcomes (eGFR changes), Complications, Conversion to radical nephrectomy, Blood transfusion rates	Off-clamp RAPN does not result in better renal functional preservation compared to on-clamp. Off-clamp was associated with increased rates of conversion to radical nephrectomy (10.2% vs 1%) and need for blood transfusion (2.9% vs 0%, p=0.030). Mean fall in eGFR at last follow-up was equivalent between groups.	Robot-assisted partial nephrectomy, Complete data on type of ischemia, Small renal masses (partial nephrectomy candidates)	Inadequate data, Patients without complete data.
			Off-clamp RPN										
12	Rac et al. (2024) [25]	2024	On-clamp RAPN	USA	Retrospective comparative study	March 2012 - April 2022	189	Retrospective	Single-center	1. Perioperative outcomes (surgical time, blood loss, complications, hospital length of stay) 2. Oncologic outcomes (recurrence rates, positive margins) 3. Renal functional outcomes (eGFR changes at POD1, discharge, 3 months, 6-9 months, 12 months, and last follow-up)	There is no significant difference in perioperative outcomes such as surgical time, blood loss, or complications between the two groups. Furthermore, there was no significant difference in postoperative kidney function between the two techniques.	Patients who underwent robotic-assisted laparoscopic partial nephrectomy via tumor enucleation- Surgery performed by a single surgeon- Surgery between March 2012 and April 2022- Preoperative creatinine data available	Patients who underwent conversion to standard margin PN- Patients who underwent conversion to radical nephrectom- Patients who underwent conversion to open surgical procedure - Patients for whom preoperative creatinine data were not available (preoperative GFR could not be calculated)
			Off-clamp RAPN										
13	Guo et al. (2019) [38]	2019	On-clamp RRLPN	China	Retrospective comparative study	January 1, 2015 - December 31, 2017	93	Retrospective	Single-center	1. Perioperative outcomes (operative time, tumor resection time, warm ischemia time, estimated blood loss, hospital stay, transfusion rate) 2. Renal functional outcomes (preoperative ipsilateral GFR, postoperative 6-months ipsilateral GFR, ΔGFR6) 3. Complications (Clavien-Dindo classification) 4. Oncologic outcomes (positive surgical margin, postoperative recurrence)	Off-clamp robotic partial nephrectomy via retroperitoneal approach is a safe and effective technique for the removal of renal tumor while the indication of surgery is strictly limited to small (<4 cm) and exophytic renal tumor.	Patients underwent robot-assisted retroperitoneal laparoscopic partial nephrectomy (RRLPN) - Surgery performed by a single surgical team - Surgery between January 1, 2015 and December 31, 2017 - Tumors confirmed by CT or MRI to be exophytic - Tumor diameter <4 cm	Patients who underwent conversion to standard margin PN - Patients who underwent conversion to radical nephrectomy- Patients who underwent conversion to open surgical procedure - Preoperative creatinine data not available
			Off-clamp RRLPN										
14	Ener et al. (2016) [36]	2016	On-clamp RPN	Turkey	Retrospective	2009 - 2015	34	Retrospective	Multi-center (2 centers)	1. Perioperative outcomes (operation time, estimated blood loss, hospital stay) 2. Renal functional outcomes (serum creatinine and eGFR at preoperative, immediate postoperative, and postoperative 3rd month) 3. Complications (modified Clavien-Dindo classification system) 4. Oncologic outcomes (positive margins, recurrence)	Off-clamp RPN technique is superior, in short-term outcomes involving renal functions, compared to on clamp approach. However, long-term data regarding the renal functions should be evaluated to arrive at a definitive decision.	Patients underwent transperitoneal robotic partial nephrectomy (RPN) - Surgery performed using four-arm da Vinci-S robotic surgical system - Surgery between 2009 and 2015 - Renal mass confirmed by abdominal CT or MRI - Clinical stage T1 tumors	Not explicitly stated in the study
			Off-clamp RPN										
15	Belmonte et al. (2024) [33]	2024	On-clamp RAPN	Belgium	Retrospective cohort with IPTW	2016-2023	532 (394 on-clamp, 138 off-clamp) with IPTW sample size of Not applicable (IPTW weighted: 224 on-clamp, 86 off-clamp)	Retrospective	1 (high-volume tertiary)	Modified trifecta achievement	Clamping technique does not imply clinically relevant differences in reaching trifecta outcomes. Off-clamp technique was not a predictor of trifecta achievement after IPTW adjustment.	Patients undergoing RAPN for cT1-cT2 renal masses between 2016-2023	Patients with solitary kidney, bilateral renal masses, or incomplete clinical data
			Off-clamp RAPN										
16	Mutelica et al. (2020) [23]	2020	On-clamp RAPN	France	Retrospective matched case-control	January 2007 - December 2015	940 (837 on-clamp, 103 off-clamp) and matched sample size are 412 (309 on-clamp, 103 off-clamp)	Retrospective	1 (tertiary robotic center)	Perioperative morbidity, functional outcomes (eGFR decline, preservation, CKD upstaging)	Off-clamp RAPN for selected renal masses offered renal functional advantage over on-clamp without adding morbidities. Off-clamp approach, excisional volume loss, and age were independent predictors of renal function preservation.	Patients undergoing RAPN for cT1a tumors between 2007-2015	Patients with solitary kidney or multifocal tumors
			Off-clamp RAPN										
17	Peyronnet et al. (2017) [25]	2017	On-clamp RAPN	France	Match-Paired Case–Control Study	January 2010 - December 2014	525 (499 on-clamp, 26 off-clamp) and with matching 130 (104 on-clamp, 26 off-clamp)	Retrospective	8 (academic departments)	Perioperative outcomes, postoperative renal function	Off-clamp RPN is feasible for selected renal masses without increasing complications but at cost of higher blood loss and increased risk of conversion to radical nephrectomy.	Patients undergoing RPN at 8 academic centers between 2010-2014	Patients who underwent RPN with selective arterial clamping
			Off-clamp RAPN										
18	Bertolo et al. (2019) [34]	2019	On-clamp RAPN	Multi-national (USA - Cleveland Clinic; Italy - Regina Elena National Cancer Institute)	Retrospective, Propensity-Score-Matched (2:1), Bi-centric Comparative Study	January 2007 - December 2017	1983 total patients (Overall: 1389 on-clamp from Cleveland Clinic, 471 off-clamp from Regina Elena; PSM: 400 on-clamp, 200 off-clamp). 123 off-clamp patients from Cleveland Clinic excluded to ensure each institution contributed unselected patients	Retrospective	Multi-center (2 high-volume institutions)	Perioperative outcomes; Long-term functional outcomes (eGFR at baseline, discharge, 12 and 24 months); Oncological outcomes (recurrence-free survival, metastasis-free survival, overall survival, cancer-specific survival)	No significant differences in perioperative and oncological outcomes between off-clamp and on-clamp RAPN. Avoided ischemia benefits renal function within 1-year follow-up after surgery (5 mL/min difference at 12 months). At longer follow-up (24 months), difference with on-clamp is softened (2 mL/min difference), possibly due to hyperfiltration in on-clamp group	Patients who underwent off-clamp or on-clamp (warm ischemia) RAPN for renal tumors suitable for partial nephrectomy, with propensity matching based on age, sex, smoking, diabetes, hypertension, ASA score, solitary kidney status, preoperative eGFR (calculated by CKD-EPI), tumor size, and R.E.N.A.L. score; mean age ~60 years with baseline eGFR >60 mL/min/1.73m² in majority of patients	123 "selected" off-clamp cases from Cleveland Clinic were excluded to ensure unselected patient cohorts from each institution; solitary kidney NOT excluded (2.5-3.5% had solitary kidney); bilateral/multiple tumors likely excluded (not specified); previous renal surgery not specified; baseline CKD NOT excluded (various CKD stages included)
			Off-clamp RAPN										
19	AnCeSChi et al. (2022) [19]	2022	On-clamp RAPN	Multi-national (Italy - 6 centers: Regina Elena Institute Rome, Careggi Hospital Florence, San Raffaele Hospital Milan, San Luigi Gonzaga Hospital Turin; USA - 2 centers: Virginia Commonwealth University, Temple University Philadelphia)	Retrospective, Propensity-Score-Matched (1:1), Multi-institutional Collaborative Study	July 2007 - March 2021	222 total patients ≥75 years (Overall: 113 on-clamp, 109 off-clamp initially; After exclusions: PSM: 104 on-clamp, 101 off-clamp)	Retrospective	Multi-center (6 tertiary-care centers: 4 Italy, 2 USA)	Primary: Predictors of severe CKD (sCKD stages ≥3b); Secondary: Comparison of perioperative and functional outcomes between on-clamp and off-clamp RAPN in elderly patients	RPN in patients ≥75 years is a safe surgical option. On-clamp approach (HR 3.41), hypertension (HR 2.64), and non-achievement of trifecta (HR 0.36) were independent predictors of sCKD in elderly after RPN. Off-clamp associated with significantly lower risk of developing sCKD (p=0.002 at Kaplan-Meier)	Elderly patients aged ≥75 years (mean 78 years, IQR 76-80) who underwent elective RAPN for clinical T1-T2 contrast-enhancing renal masses (mean tumor size 2.3 cm, mean R.E.N.A.L. score 5.7±1.9) on CT/MRI, with propensity matching based on tumor nephrometry score and preoperative renal functional classification; median baseline eGFR 66.1 mL/min/1.73m² with 29.8% having baseline CKD stage ≥3a	Solitary kidneys (9 patients EXCLUDED), bilateral/multiple renal masses (1 patient EXCLUDED), missing data (7 patients EXCLUDED), and 5 patients from each cohort excluded from functional analysis due to unavailable creatinine measurements (functional analysis: 24 per group); previous renal surgery not specified; baseline CKD NOT excluded (all CKD stages included)
			Off-clamp RAPN										
20	Tanagho et al. (2012) [28]	2012	On-clamp RAPN	USA (Washington University School of Medicine, St Louis, Missouri)	Retrospective, Propensity-Score-Matched (1:1) Cohort Study	March 2008 - September 2011	164 total patients (Overall: 135 on-clamp, 29 off-clamp; PSM: 29 on-clamp matched to 29 off-clamp)	Retrospective	Single-center	Renal functional outcomes (postoperative changes in eGFR at mean 9-month follow-up); Perioperative safety profile (EBL, operative time, complications)	Off-clamp RAPN is associated with favorable morbidity profile and relatively greater renal functional preservation compared to clamped RAPN (decline of 4.9 vs 11.7 mL/min/1.73m², p=0.033). Nevertheless, the benefit is small in renal functional terms and may have very limited clinical relevance	Patients with suspected renal cell carcinoma showing contrast-enhancing renal masses on preoperative CT/MRI who underwent elective RAPN, matched by identical R.E.N.A.L. nephrometry scores and comparable baseline renal function (eGFR by CKD-EPI formula) using National Kidney Foundation Classification; mean age ~60 years with all treatment options including surveillance discussed	Five patients from each matched cohort excluded from functional outcomes analysis due to unavailable baseline and/or postoperative creatinine measurements (total functional analysis: 24 per group); solitary kidney NOT excluded (3.4% in off-clamp group); bilateral/multiple tumors not specified; previous renal surgery not specified; baseline CKD NOT excluded (various CKD stages included)
			Off-clamp RAPN										

Quality Assessment

**Figure 2:**
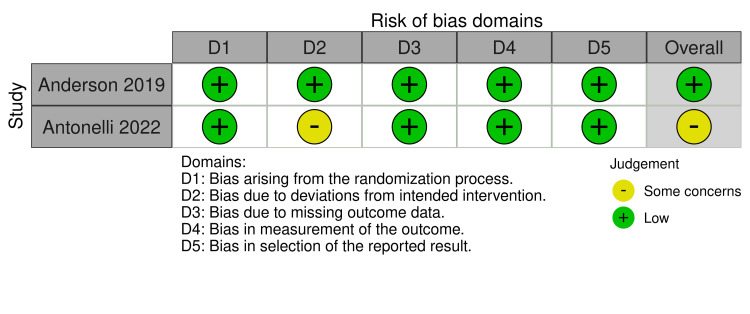
Risk of Bias Assessment References [31,32]

NOS assessment of the 18 observational studies revealed that 15 studies (83.3%) demonstrated good methodological quality. One study (5.6%), Güner et al. (2021), was rated as fair quality due to inadequate comparability adjustment. Two studies (11.1%), Sharma et al. (2023) and Peyronnet et al. (2017), were rated as poor quality due to insufficient comparability adjustment and inadequate follow-up documentation, respectively. Most studies performed well in the selection and outcome domains, while 15 studies (83.3%) adequately controlled for confounding factors through propensity score matching or multivariate adjustment. Both RCTs demonstrated a low overall risk of bias. The detailed data are presented in Table 3. Anderson et al. (2019) showed low risk across all domains. Antonelli et al. (2022) also demonstrated a low risk across most domains, though some concerns were raised about deviations from the intended intervention. Detailed presentations are shown in Figure 2.

**Table 3:** Newcastle-Ottawa Scale (NOS) Assessment

ID	New Castle Ottawa scale assessment (NOS)								
	Cohort studies								
	Selection				Comparability	Outcome			Quality Score
	Representativeness of the exposed cohort	Selection of the non exposed cohort	Ascertainment of exposure	Demonstration that outcome of interest was not present at start of study	Comparability of cohorts on the basis of the design or analysis	Assessment of outcome	Was follow-up long enough for outcomes to occur	Adequacy of follow up of cohorts	
Brassetti et al.(2022) [35]		*	*	*	*	*	*	*	Good
Mari et al. (2018) [22]		*	*	*	**	*	*		Good
Anderson et al. (2018) [32]	*	*	*	*	*	*		*	Good
Kaczmarek et al. (2013) [21]	*	*	*	*	**	*	*	*	Good
Rosen et al. (2017) [26]	*	*	*	*	**	*	*		Good
Güner et al. (2021) [37]	*	*	*	*		*	*	*	Fair
Vargo et al. (2024) [28]		*	*	*	**	*		*	Good
Tuderti et al. (2023) [29]	*	*	*	*	**	*	*	*	Good
Sharma et al. (2023) [27]	*	*	*	*		*	*	*	Poor
Rac et al. (2024) [25]	*	*	*	*	*	*	*	*	Good
Guo et al. (2019) [38]	*	*	*	*	*	*		*	Good
Ener et al. (2016) [36]	*	*	*	*	*	*	*		Good
Belmonte et al. (2024) [33]	*	*	*	*	**	*		*	Good
Mutelica et al. (2020) [23]		*	*	*	**	*	*		Good
Peyronnet et al. (2017) [24]		*	*	*	*	*			Poor
Bertolo et al. (2019) [34]	*	*	*	*	**	*	*	*	Good
AnCeSChi et al. (2022) [19]	*	*	*	*	**	*	*		Good
Tanagho et al. (2012) [28]	*	*	*	*	**	*	*		Good

Efficacy outcomes

Glomerular Filtration Rate Change

Analysis of postoperative GFR changes included 18 studies with 2,629 patients in the on-clamp group and 1,657 patients in the off-clamp group. The pooled MD was -3.45 mL/min/1.73m^2^ (95% CI: (-5.05 to -1.85); p = 0.0001), indicating a statistically significant difference favoring the off-clamp technique. High heterogeneity was present (I^2 ^= 87.8%, p < 0.0001). Renal function outcomes across the included studies were heterogeneous and couldn’t be resolved through any sensitivity analysis.

**Figure 3:**
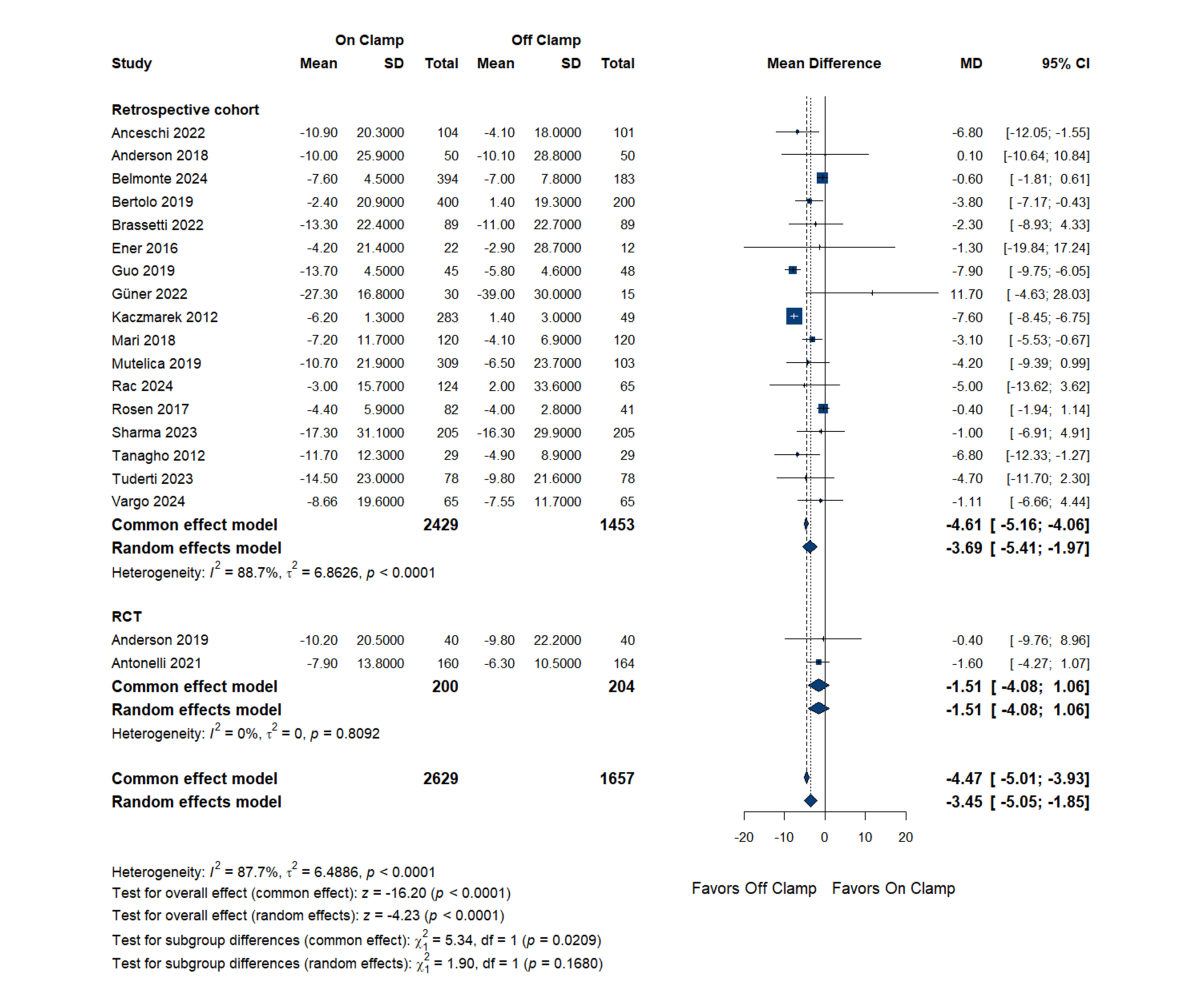
Glomerular Filtration Rate Change (Subgroup Analysis by Study Design) RCT: randomized controlled trial, SD: standard deviation, MD: mean difference, CI: confidence interval References [14,16-21,23-27,29-33,35]

Subgroup analysis by study design: Stratified by study design, subgroup analysis revealed differences between study types. Retrospective cohort studies (17 studies) showed a pooled MD of -3.69 mL/min/1.73 m^2^ (95% CI: -5.41 to -1.97; p < 0.001), with high heterogeneity (I^2 ^= 88.7%). RCTs (two studies) demonstrated a pooled MD of -1.51 mL/min/1.73 m^2^ (95% CI: -4.08 to 1.06; p = 0.809), with no heterogeneity (I^2 ^= 0%). The test for subgroup differences showed no significant difference between RCTs and observational studies (p = 0.168) (Figure 3).

**Figure 4:**
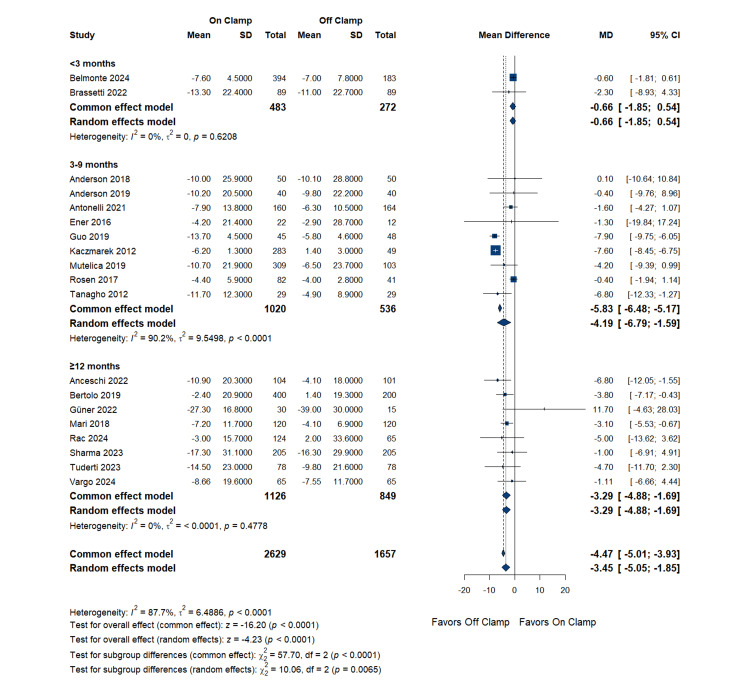
Glomerular Filtration Rate Change (Subgroup Analysis by Follow-Up Duration) RCT: randomized controlled trial, SD: standard deviation, MD: mean difference, CI: confidence interval References [14,16-21,23-27,29-33,35]

Subgroup analysis by follow-up duration: Studies were categorized into three groups: less than three months (two studies), three to nine months (nine studies), and ≥12 months (eight studies). The less than three months subgroup showed a pooled MD of -0.66 mL/min/1.73 m^2^ (95% CI: -1.85 to 0.54; p = 0.621; I^2 ^= 0%). The three to nine months subgroup demonstrated a pooled MD of -4.19 mL/min/1.73m^2^ (95% CI: -6.79 to -1.59; p < 0.001, I^2 ^= 90.2%). The ≥12 months subgroup showed a pooled MD of -3.29 mL/min/1.73 m^2^ (95% CI: -4.88 to -1.69; p < 0.001; I^2 ^= 0%) (Figure 4).

**Figure 5:**
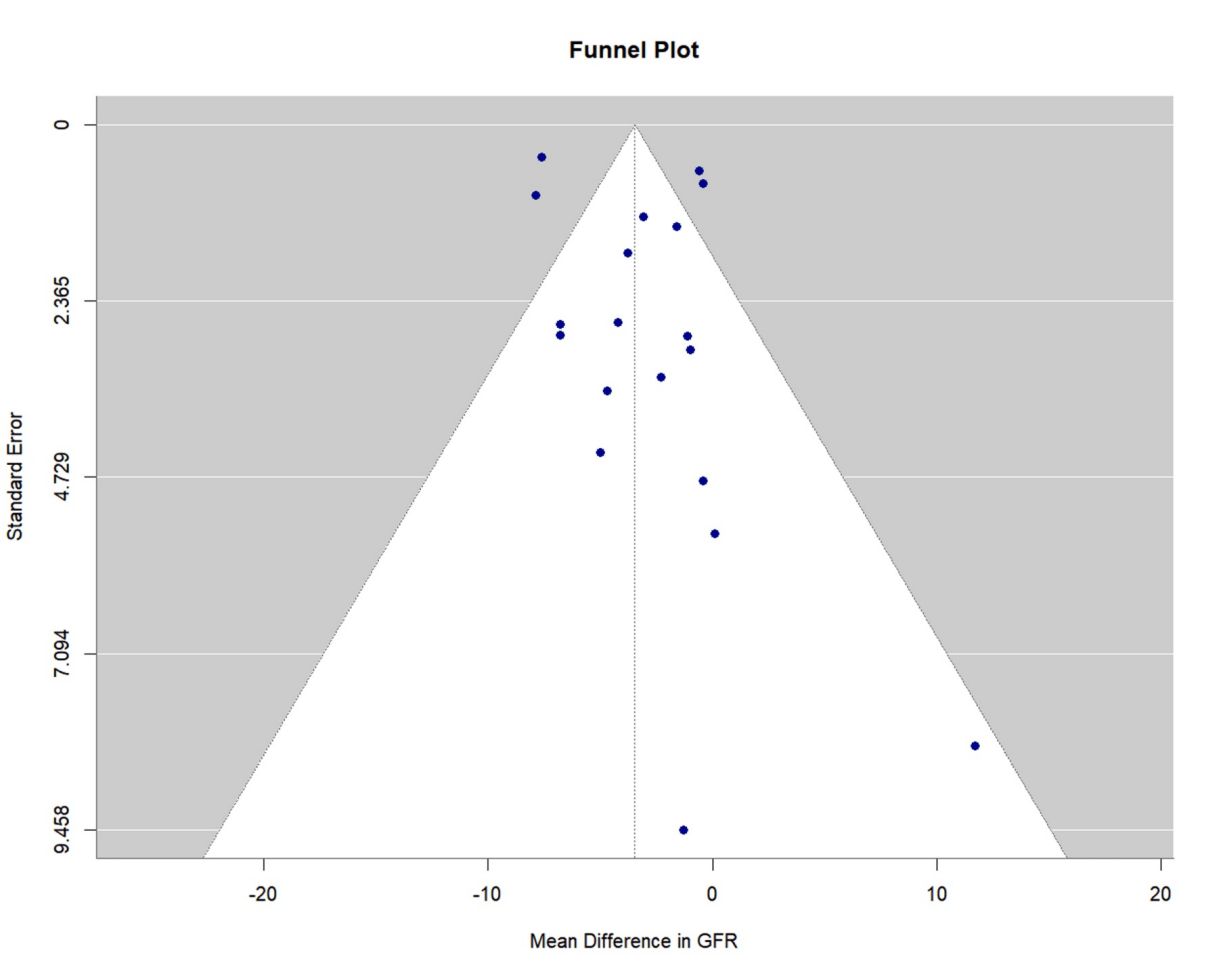
Funnel Plot for Glomerular Filtration Rate Change

Publication bias assessment: Visual inspection of the funnel plot revealed relative symmetry with most studies distributed within the expected confidence interval boundaries. Egger's regression test showed no evidence of significant publication bias (bias estimate = 1.07, standard error (SE) = 0.96, p = 0.278). The funnel plot is presented as Figure 5.

Positive Surgical Margins

**Figure 6:**
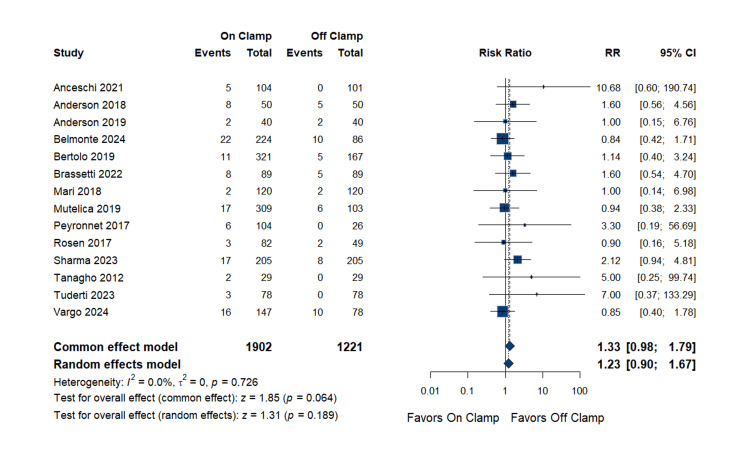
Positive Surgical Margins RR: risk ratio, CI: confidence interval References [14,17,18,20-22,24-27,29,31-33]

Analysis of positive surgical margins included 14 studies with 1,902 patients in the on-clamp group and 1,221 patients in the off-clamp group. The pooled RR was 1.23 (95% CI: 0.90 to 1.67; p = 0.189), showing no statistically significant difference between the two techniques. The data were homogenous (p = 0.726; I^2^ = 0.0%). Complete data are in Figure 6.

**Figure 7:**
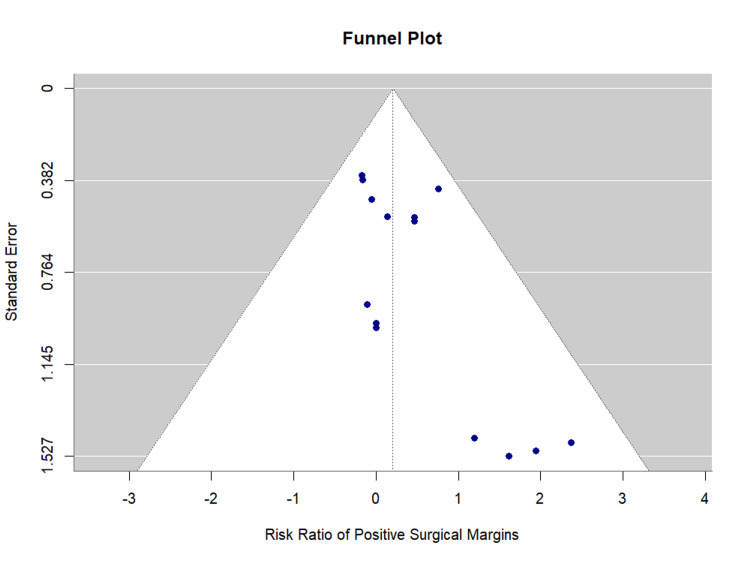
Funnel Plot for Positive Surgical Margins

Publication bias assessment: Egger's regression test indicated evidence of small-study effects (p = 0.026), suggesting potential publication bias. However, given the homogeneity of the analysis (I^2^ = 0%) and the non-significant pooled estimate, the clinical impact appears limited. The funnel plot is presented as Figure 7.

Safety outcomes

Major Complications

**Figure 8:**
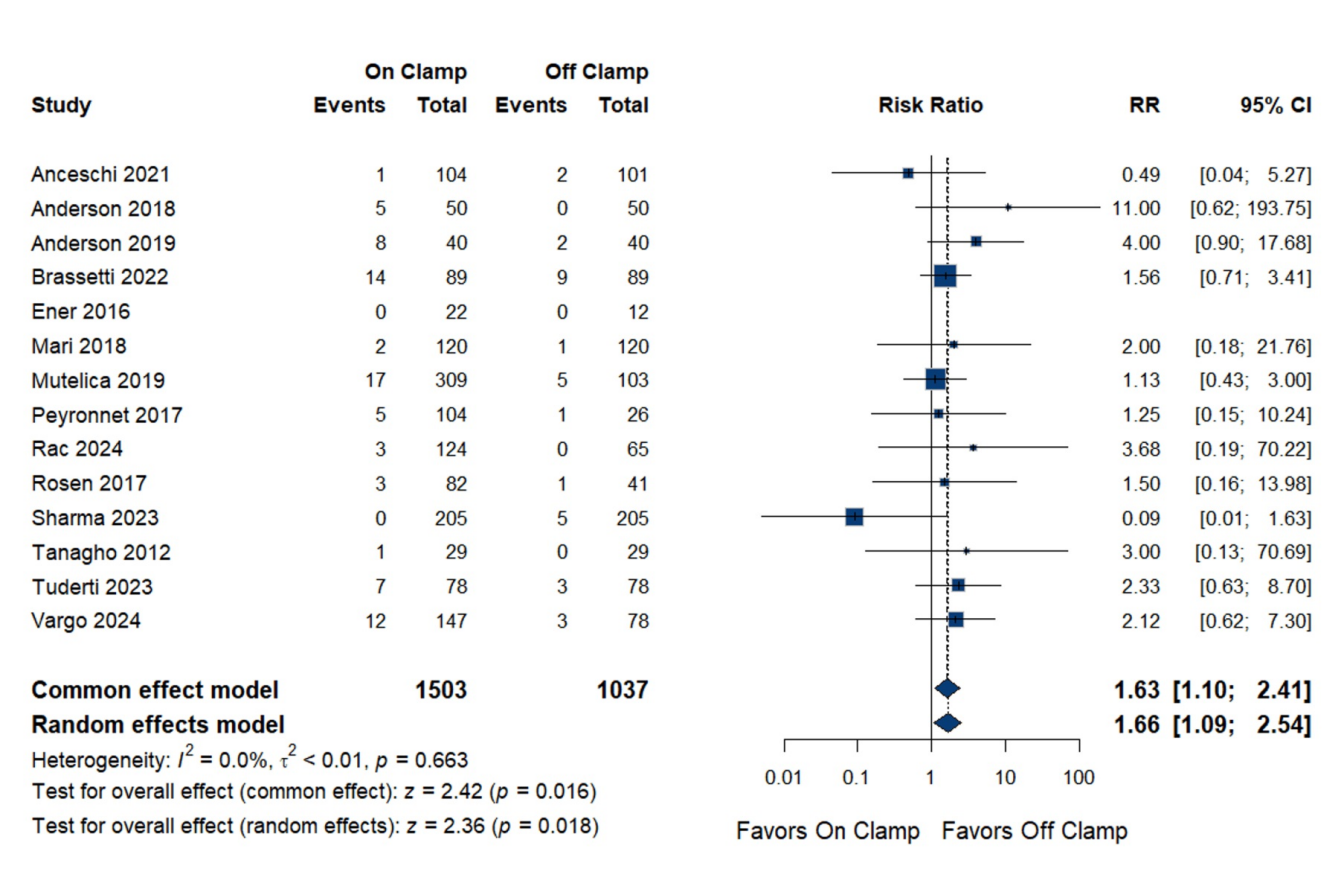
Major Complications RR: risk ratio, CI: confidence interval References [14,17-23,25-27,29,32,33]

The analysis of major complications included 14 studies with a total population of 1,503 in the on-clamp group and 1,037 in the off-clamp group. The pooled RR was 1.66 (95% CI: (1.09 to 2.54); p = 0.018), indicating a statistically significant increased risk of major complications in the on-clamp group compared to the off-clamp group. The analysis showed no heterogeneity (p = 0.663; I^2^ = 0%). Complete data are presented in Figure 8.

**Figure 9:**
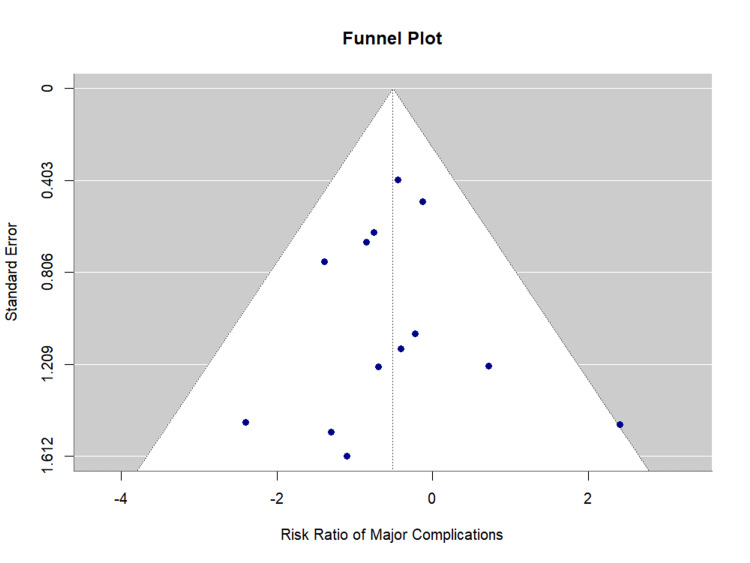
Funnel Plot for Major Complications

Publication bias assessment: Egger's regression test showed no evidence of significant publication bias (p = 0.946). The funnel plot is presented as Figure 9.

Blood Transfusion Requirements

**Figure 10:**
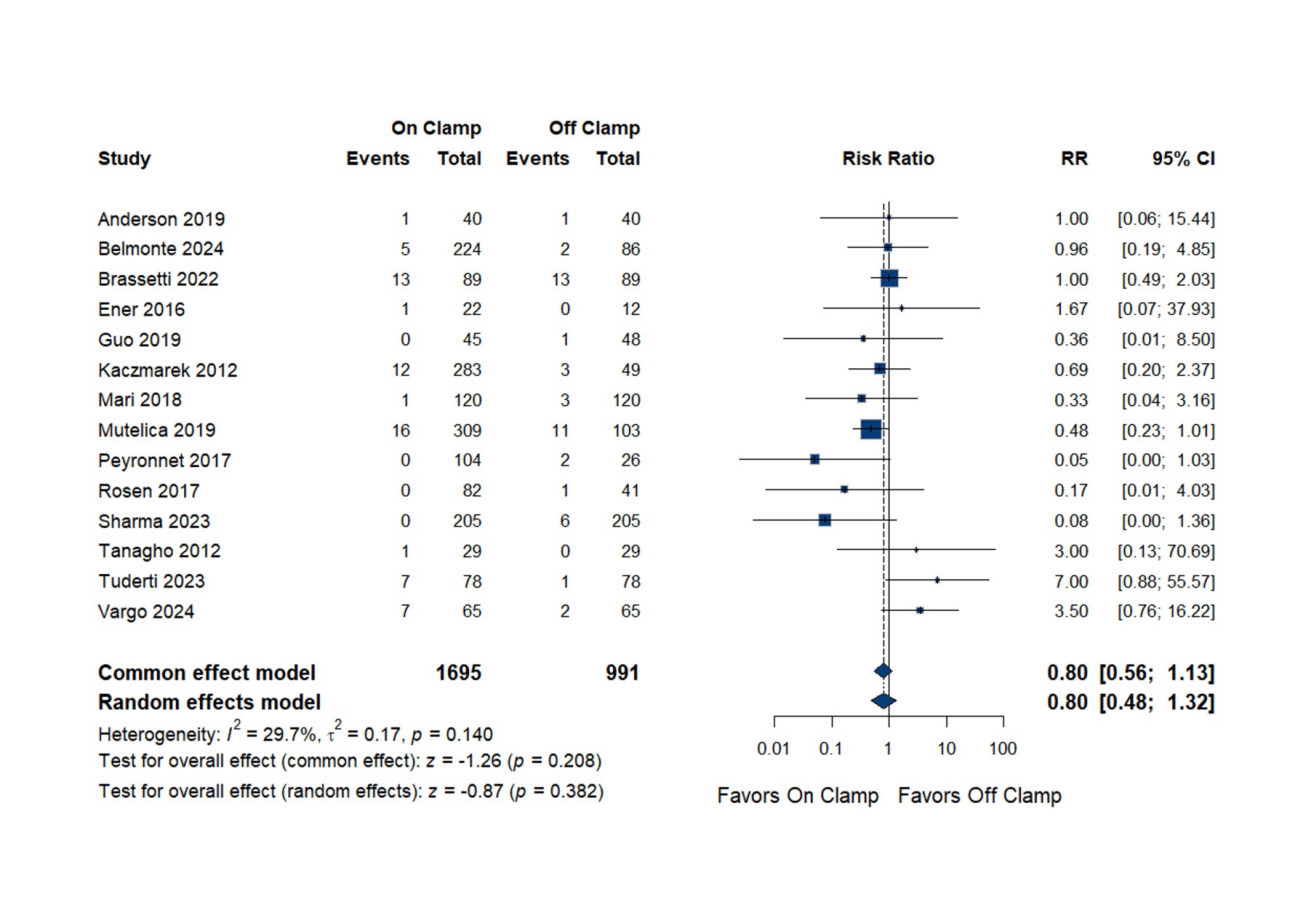
Blood Transfusion Requirements RR: risk ratio, CI: confidence interval References [14,16,18-22,25-27,29,31-33]

The meta-analysis of 14 studies comprising 1,695 patients in the on-clamp group and 991 patients in the off-clamp group revealed no statistically significant difference in blood transfusion requirements. The pooled RR was 0.80 (95% CI: (0.48 to 1.32); p = 0.382), suggesting similar transfusion rates between the two techniques. The data were homogenous (p = 0.140; I^2^ = 29.7%). Complete data are presented in Figure 10.

**Figure 11:**
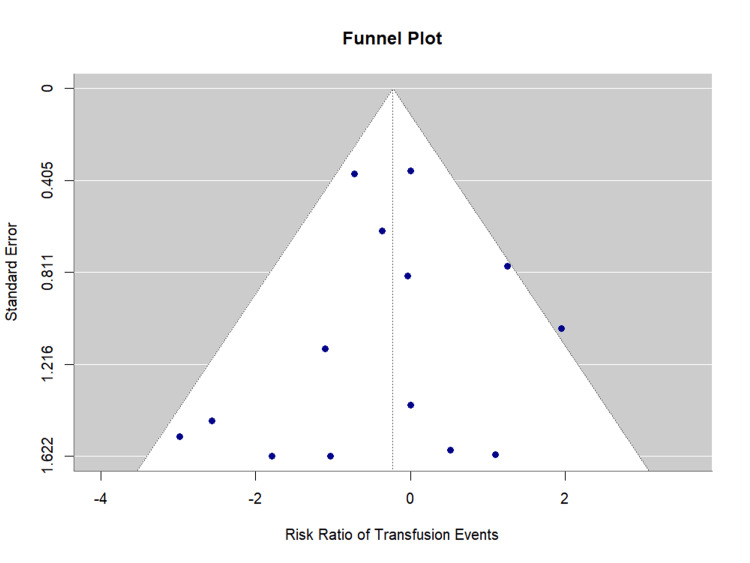
Funnel Plot for Blood Transfusion Requirements

Publication bias assessment: Egger's regression test showed no evidence of significant publication bias (p = 0.800). The funnel plot is presented as Figure 11.

Perioperative outcomes

Estimated Blood Loss

**Figure 12:**
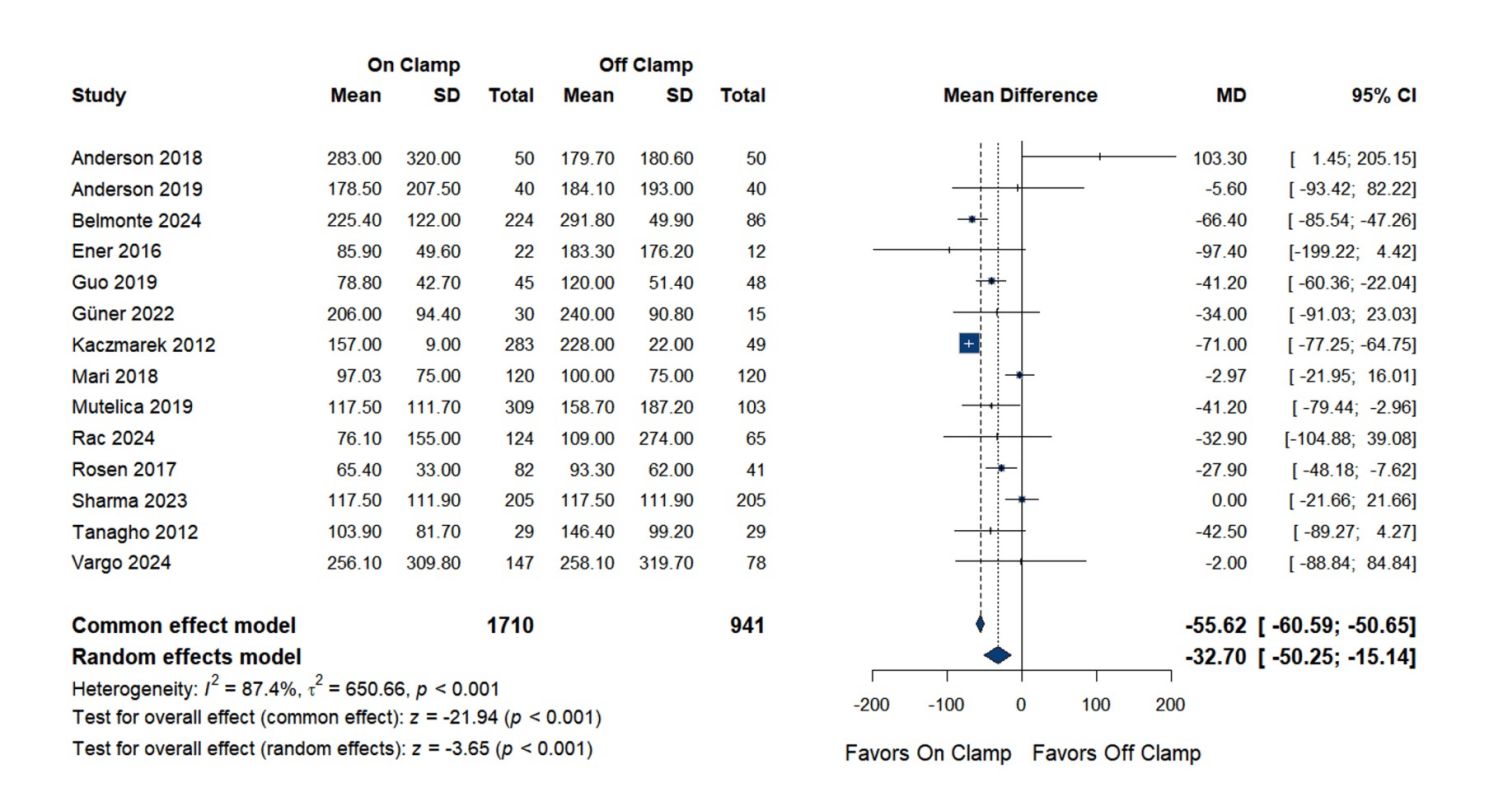
Estimated Blood Loss SD: standard deviation, MD: mean difference, CI: confidence interval References [14,16,18-21,23,25-27,29,31,32,35]

The 14 studies, involving 1,710 patients in the on-clamp group and 941 in the off-clamp group, demonstrated a statistically significant difference in estimated blood loss. The pooled MD was -32.7 mL (95% CI: (-50.3 to -15.1); p < 0.001), favoring the on-clamp technique. However, substantial heterogeneity was observed among the included studies (I^2^ = 87.4%, p < 0.0001), and it couldn’t be resolved with any sensitivity analysis. Complete data are presented in Figure 12.

**Figure 13:**
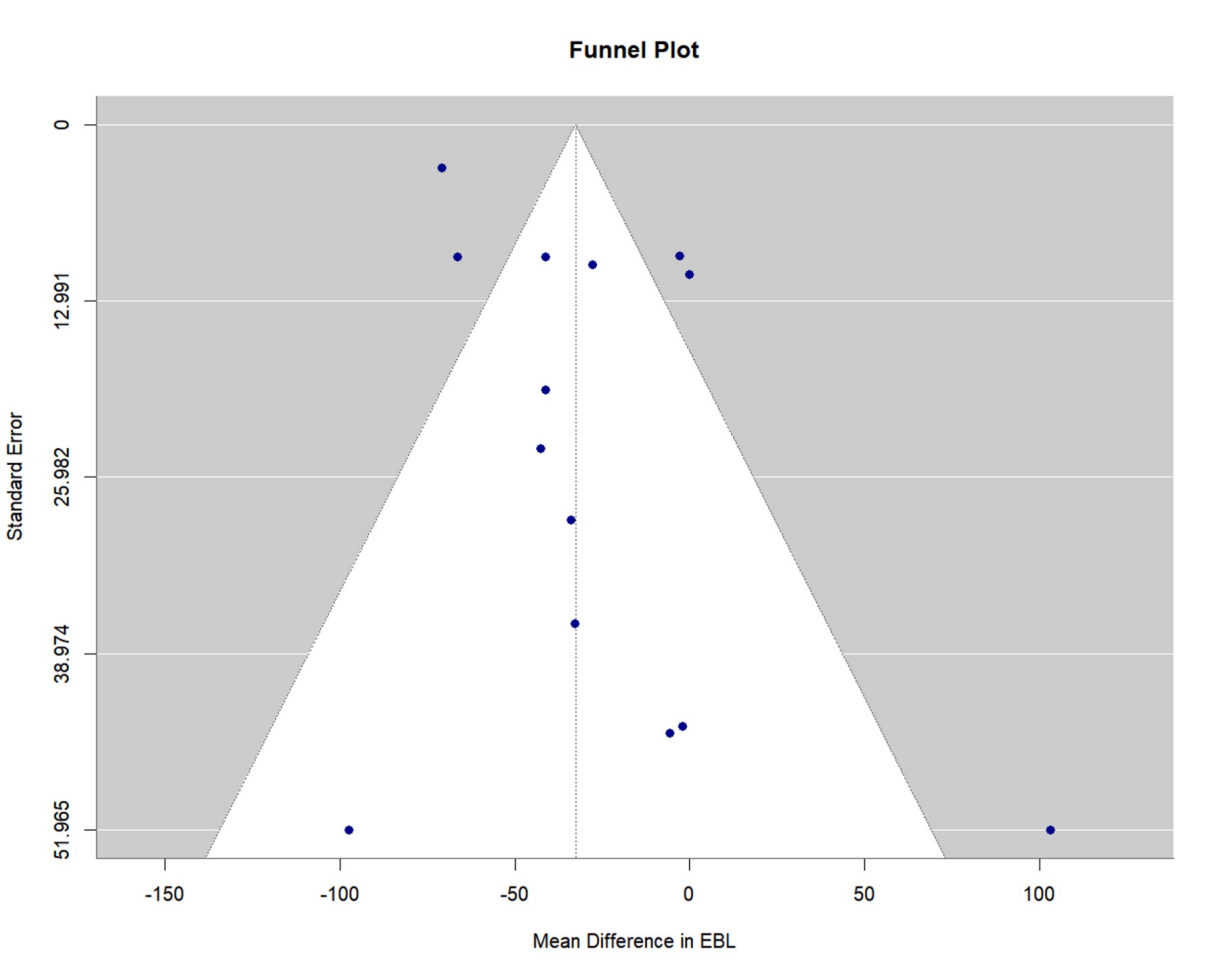
Funnel Plot for Estimated Blood Loss

Publication bias assessment: Egger's regression test indicated evidence of small-study effects (p = 0.025), suggesting potential publication bias. However, the finding of reduced blood loss with on-clamp remained statistically significant despite this bias. The funnel plot is presented as Figure 13.

Hospital Stay Duration

**Figure 14:**
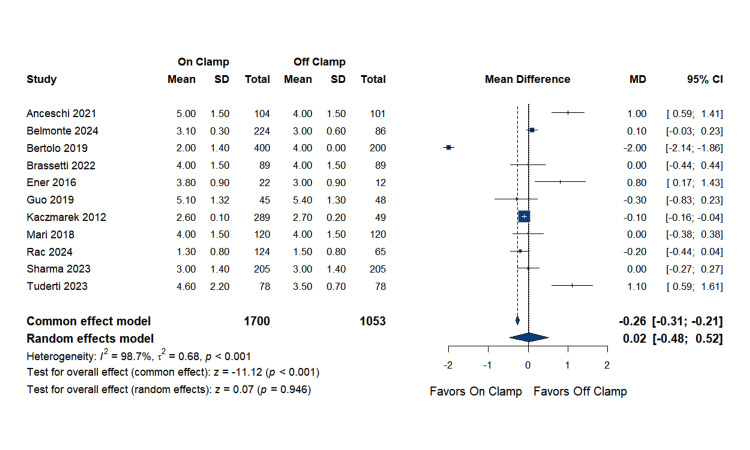
Hospital Stay Duration SD: standard deviation, MD: mean difference, CI: confidence interval References [16,17,19,20,23-25,27,31,33]

The pooled analysis of 11 studies comprising 1,700 patients in the on-clamp group and 1,053 patients in the off-clamp group revealed no statistically significant difference between the two techniques. The MD was 0.02 days (95% CI: (-0.48 to 0.52); p = 0.9462). Substantial heterogeneity was noted (I^2^ = 98.7%). Complete data are presented in Figure 14.

**Figure 15:**
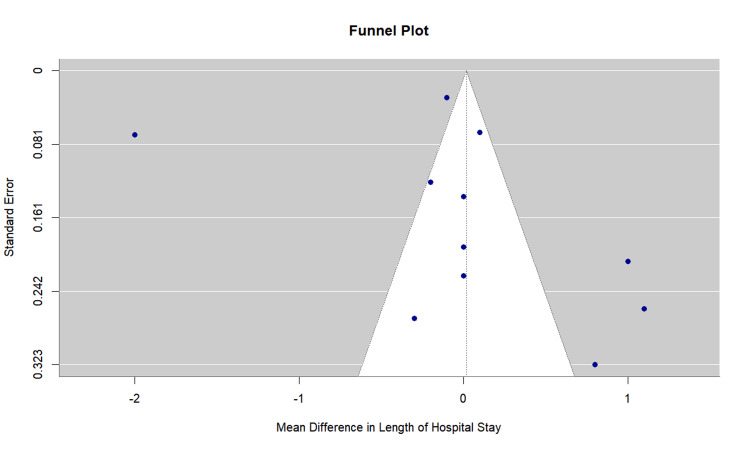
Funnel Plot for Hospital Stay Duration

Publication bias assessment: Egger's regression test showed no evidence of significant publication bias (p = 0.806). The funnel plot is presented in Figure 15.

Operative Time

**Figure 16:**
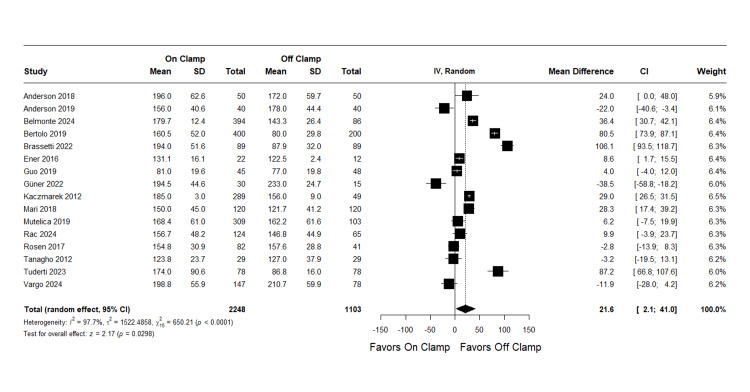
Operative Time SD: standard deviation, MD: mean difference, CI: confidence interval References [14,16,18-21,23,24,26,27,29,31-33,35]

The meta-analysis included 16 studies with 2,248 patients in the on-clamp group and 1,103 in the off-clamp group. The results showed a pooled MD of 21.55 minutes (95% CI: (2.11 to 40.99); p = 0.03), favoring the off-clamp technique. However, considerable heterogeneity was present (I^2^ = 97.7%, p < 0.0001), and couldn’t be resolved with any sensitivity analysis. Complete data are presented in Figure 16.

**Figure 17:**
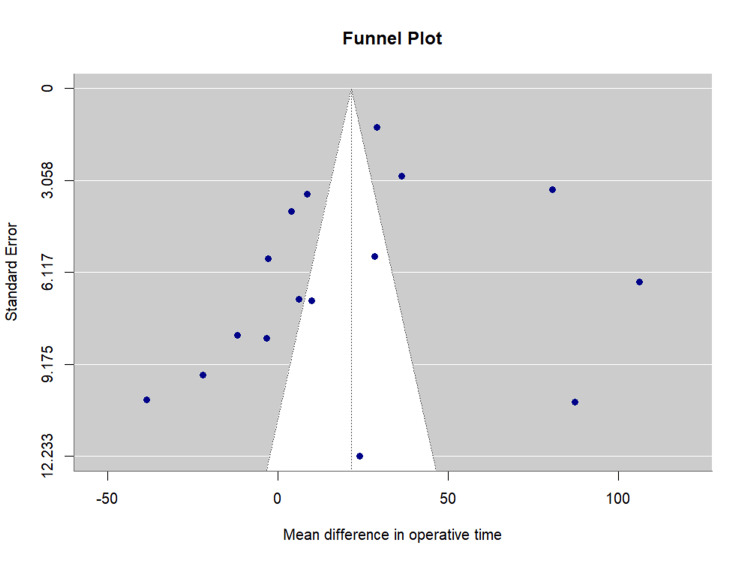
Funnel Plot for Operative Time

Publication bias assessment: Egger's regression test showed no evidence of significant publication bias (p = 0.519). The funnel plot is presented as Figure 17.

Discussion

This systematic review and meta-analysis comparing off-clamp versus on-clamp RAPN included 20 studies with 4,961 patients across multiple countries. Our analysis demonstrated that off-clamp RAPN is superior to on-clamp RAPN for major complications, warranting particular attention. Notably, this increased complication rate occurred despite the on-clamp approach demonstrating superior hemostatic control with less estimated blood loss. Paradoxically, the on-clamp approach showed less estimated blood loss, yet this did not translate into differences in transfusion requirements, suggesting the complications are primarily ischemia-related rather than hemorrhagic [39,40]. Despite greater blood loss, the off-clamp group experienced fewer major complications, suggesting that morbidity in on-clamp RAPN is more likely driven by ischemia than by hemorrhage. Ischemia-reperfusion injury, involving oxidative stress, inflammation, and microvascular dysfunction, may explain this trend and its associated postoperative risks.

Consistent with this, renal function outcomes favored the off-clamp approach, showing significantly better GFR preservation (-3.45 mL/min/1.73m^2^), though the clinical impact remains modest [39]. Subgroup analysis by follow-up duration revealed that this benefit was most pronounced at intermediate (three to nine months) and long-term (≥12 months) follow-up, with minimal difference observed in the immediate postoperative period (less than three months). Notably, the long-term data (≥12 months) demonstrated sustained benefit with low heterogeneity (I^2^ = 0%), suggesting reliable and consistent findings across studies. Preservation of renal function represents a primary advantage of PN over radical nephrectomy, with postoperative renal outcomes influenced not only by patient and tumor characteristics but also by modifiable intraoperative technical factors, including resection method, renorrhaphy approach, and renal pedicle management strategies [41,42]. However, the clinical impact of the observed GFR difference remains modest and may be limited in patients with normal baseline renal function. This difference may prove more meaningful in high-risk subgroups with solitary kidneys, pre-existing CKD, or complex tumors requiring prolonged resection time [40].

From an oncological perspective, both techniques showed comparable efficacy with no significant difference in positive surgical margin rates, indicating adequate tumor control regardless of clamping approach. Several studies reported long-term oncological outcomes inconsistently, precluding meaningful meta-analysis. Anderson et al. identified one patient who developed metastatic disease without specifying the treatment group [31]. Mari et al. documented three total recurrences, with one occurring in the off-clamp group and two in the on-clamp group [22]. Bertolo et al. reported comparable rates of mortality, recurrence, and metastasis between the approaches [34]. Peyronnet et al. observed one recurrence in the on-clamp arm with no recurrences in the off-clamp arm [24].

Regarding perioperative efficiency, off-clamp RAPN required a significantly longer operative time, likely due to the technical complexity of achieving hemostasis without vascular occlusion. However, hospital stay duration was equivalent between approaches, indicating similar recovery trajectories and suggesting that longer operative time does not adversely impact postoperative recovery [40].

Our findings both align with and diverge from previous meta-analyses examining off-clamp versus on-clamp RAPN. The most recent meta-analysis by Shrivastava et al. (2023) included 11 studies involving 2,483 patients. They concluded that there were no clinically relevant differences in perioperative and functional outcomes between techniques. In contrast, our study demonstrates a statistically significant increased risk of major complications with on-clamp RAPN. While Shrivastava et al. found no difference in estimated blood loss, our larger dataset showed significantly less blood loss with on-clamp [13]. However, both studies agreed that this did not affect transfusion requirements. Shrivastava et al. reported significantly lower rates with off-clamp, while our analysis found no significant difference. Both studies concur on equivalent hospital stay duration and operative time, showing no consistent advantage for either approach after sensitivity analyses [13].

An additional similar meta-analysis, Fong et al., 2024, analyzed 10 studies involving 2,307 patients. They demonstrated that both off-clamp and on-clamp RAPN are similarly effective, with off-clamp possibly offering better renal function preservation and reduced margin-positive rates. While Fong et al. found no significant difference in estimated blood loss between techniques, our larger dataset revealed significantly less blood loss with on-clamp. Both studies agreed that the off-clamp technique provided superior eGFR preservation, though the clinical significance remains debatable [12]. Regarding positive surgical margins, Fong et al. reported significantly lower rates with off-clamp, while our analysis found no significant difference. Both studies concur on equivalent hospital stay duration, operative time, and transfusion requirements, showing no consistent advantage for either approach [12].

This meta-analysis possesses several notable strengths that enhance the validity and clinical applicability of our findings. Firstly, we focus on studies that use RAPN to clamp the main artery, which exclude confounding from different surgical modalities and selective clamping techniques. Second, our prospectively registered protocol (PROSPERO) includes comprehensive subgroup analyses by study design and follow-up duration, partially explaining heterogeneity in renal function outcomes. Third, the substantial sample size across multiple countries provides robust statistical power and generalizability. Fourth, a systematic assessment of publication bias revealed no significant bias for most outcomes. Fifth, the use of matched populations and the inclusion of predominantly high-quality studies strengthen the internal validity of our findings.

Despite these strengths, several important limitations must be acknowledged. The substantial heterogeneity across multiple outcomes represents a significant limitation that persisted despite our sensitivity analyses and could not be fully resolved. Although propensity score matching was employed in the majority of observational studies to balance baseline covariates, residual confounding from unmeasured variables cannot be entirely excluded. The included studies didn’t specifically differentiate ischemia-related complications from hemorrhagic complications. Follow-up duration varied considerably, ranging from 90 days to three years postoperatively. Long-term oncological outcomes, including local recurrence, metastasis, and cancer-specific survival, were sparsely and inconsistently reported across studies and couldn’t be assessed in the meta-analysis. Additionally, granular data on tumor complexity stratification using validated nephrometry scoring systems (RENAL or PADUA scores) were inconsistently reported across studies. Finally, publication bias was detected for positive surgical margins and estimated blood loss, though the overall clinical conclusions remained robust.

Based on the findings and limitations of this meta-analysis, several recommendations for future research emerge. Future studies should prioritize adequately powered RCTs with standardized protocols across multiple centers. Long-term follow-up of at least five years is essential to assess CKD progression, cardiovascular outcomes, and overall survival. Studies should stratify by tumor complexity using validated nephrometry scoring systems and by baseline renal function to identify which patients derive the greatest benefit from each approach. Development of predictive models or clinical decision-support tools that incorporate patient factors, tumor characteristics, and surgeon experience would facilitate individualized surgical planning and shared decision-making. Establishment of prospective multi-institutional registries with standardized data collection would enable real-world outcome surveillance and facilitate future individual patients to provide more definitive guidance for personalized surgical approaches.

## Conclusions

Our findings suggest that off-clamp RAPN should be preferred for patients at high risk of ischemic injury, including those with solitary kidneys, pre-existing CKD, or complex tumors requiring prolonged resection time, as it significantly reduces major complications and provides superior renal function preservation, particularly at intermediate and long-term follow-up. On-clamp RAPN may be considered when minimizing operative time is prioritized, as it provides a faster procedure. While on-clamp demonstrates less blood loss, this hemostatic advantage does not translate into reduced transfusion requirements or improved safety outcomes. Both techniques demonstrate equivalent oncological efficacy with comparable positive surgical margin rates and similar hospital stay durations, indicating that oncological outcomes should not drive technique selection. However, substantial heterogeneity was observed for several outcomes, and the predominance of observational studies limits causal inference. Subgroup analysis revealed that renal function benefits were most pronounced and consistent at long-term follow-up, while no difference was observed in the immediate postoperative period. The choice between approaches should be individualized based on patient renal reserve, tumor complexity, and surgeon experience, with the off-clamp technique offering the most favorable risk-benefit profile for the majority of patients, particularly those with compromised renal function or anatomically challenging masses.

## Appendices

**Table 4:** Detailed search strategy

Database	Number of Results	Search Query
PubMed	214	("Nephrectomy" OR nephrectomies OR heminephrectomy OR heminephrectomies OR partial nephrectomy OR nephron-sparing surgery) AND ("Robotic Surgical Procedures" OR robotic surgery OR robotic-assisted surgery OR robot-assisted surgery OR robotic surgical procedure OR robotic partial nephrectomy OR robot-assisted partial nephrectomy) AND ("Kidney Neoplasms" OR kidney cancer OR renal tumor OR renal mass OR renal neoplasm) AND ("off-clamp"OR "clampless" OR "zero ischemia" OR "no ischemia" OR "non-clamping" OR "off clamp" OR "on-clamp" OR "renal artery clamping" OR "clamping technique" OR "on clamp")
Cochrane Library	58	(Nephrectomy OR nephrectomies OR heminephrectomy OR heminephrectomies OR "partial nephrectomy" OR "nephron-sparing surgery"):ti,ab,kw AND ("Robotic Surgical Procedures" OR "robotic surgery" OR "robot-assisted surgery" OR "robotic-assisted surgery" OR "robotic partial nephrectomy" OR "robot-assisted partial nephrectomy" OR "robotic surgical procedure"):ti,ab,kw AND ("Kidney Neoplasms" OR "kidney cancer" OR "renal tumor" OR "renal mass" OR "renal neoplasm" OR "kidney neoplasm" OR "renal cancer"):ti,ab,kw AND ("off-clamp" OR "off clamp" OR clampless OR "zero ischemia" OR "no ischemia" OR "non-clamping" OR "on-clamp" OR "on clamp" OR "renal artery clamping" OR "clamping technique" OR "warm ischemia"):ti,ab,kw
Scopus	122	TITLE-ABS-KEY ( ( "Nephrectomy" OR nephrectomies OR heminephrectomy OR heminephrectomies OR partial nephrectomy OR nephron-sparing surgery ) AND ( "Robotic Surgical Procedures" OR robotic surgery OR robotic-assisted surgery OR robot-assisted surgery OR robotic surgical procedure OR robotic partial nephrectomy OR robot-assisted partial nephrectomy ) AND ( "Kidney Neoplasms" OR kidney cancer OR renal tumor OR renal mass OR renal neoplasm ) AND ( "off-clamp" OR "clampless" OR "zero ischemia" OR "no ischemia" OR "non-clamping" OR "off clamp" OR "on-clamp" OR "renal artery clamping" OR "clamping technique" OR "on clamp" ) )
Web of Science (WOS)	269	ALL=(("Nephrectomy" OR nephrectomies OR heminephrectomy OR heminephrectomies OR partial nephrectomy OR nephron-sparing surgery) AND ("Robotic Surgical Procedures" OR robotic surgery OR robotic-assisted surgery OR robot-assisted surgery OR robotic surgical procedure OR robotic partial nephrectomy OR robot-assisted partial nephrectomy) AND ("Kidney Neoplasms" OR kidney cancer OR renal tumor OR renal mass OR renal neoplasm) AND ("off-clamp"OR "clampless" OR "zero ischemia" OR "no ischemia" OR "non-clamping" OR "off clamp" OR "on-clamp" OR "renal artery clamping" OR "clamping technique" OR "on clamp"))
